# Biochemical, bioluminescence and bioreporter properties of functionally improved firefly luciferases

**DOI:** 10.1016/j.jbc.2026.113568

**Published:** 2026-09-18

**Authors:** Chadaporn Kantiwiriyawanitch, Vinutsada Pongsupasa, Pratchaya Watthaisong, Jittima Phonbuppha, Aisaraphon Phintha, Daisuke Sasaki, Thanyaporn Wongnate, Ruchanok Tinikul, Kazuki Niwa, Yoshihiro Nakajima, Yoshihiro Ohmiya, Ubolsree Leartsakulpanich, Pimchai Chaiyen

**Affiliations:** 1 School of Biomolecular Science and Engineering, Vidyasirimedhi Institute of Science and Technology (VISTEC), Rayong, Thailand; 2 Health and Medical Research Institute, National Institute of Advanced Industrial Science and Technology (AIST), Takamatsu, Kagawa, Japan; 3 Faculty of Science, Department of Biochemistry and Center for Excellence in Protein and Enzyme Technology, Mahidol University, Bangkok, Thailand; 4 National Metrology Institute of Japan, National Institute of Advanced Industrial Science and Technology (AIST), Tsukuba, Ibaraki, Japan; 5 National Center for Genetic Engineering and Biotechnology (BIOTEC), National Science and Technology Agency (NSTDA), Pathum Thani, Thailand

**Keywords:** firefly luciferase, bioluminescence, enzyme engineering, reporter gene, cell signaling, real-time monitoring

## Abstract

Firefly luciferase (Fluc) is one the most widely used bioluminescent reporters for biological imaging and gene expression analysis, yet its limited stability and high assay cost remain major constraints limiting broader applications. Here, we engineered Fluc variants (N116H, M118L and N116H/M118L) with improved catalytic performance, thermostability, and soluble expression. The N116H variant exhibited the most pronounced enhancement, including a 4.6-fold increase in *k*_cat_, a 2.7-fold higher soluble expression yield, a 3.5-fold longer half-life at 37 °C, and 1.25-fold enhanced quantum yield relative to wild-type Fluc (WT). Notably, N116H required substantially less coenzyme A (CoA) to mitigate inhibition by the dehydroluciferyl-adenylate intermediate, enabling significant reduction of assay costs. Leveraging these properties, we developed a low-cost dual-luciferase reporter platform using Fluc variants and flavin-based bacterial luciferase from *Vibrio campbellii* (FLUX^Vc^) as a substitute for the conventional Fluc/Renilla luciferase (Rluc) system. The engineered systems generated comparable reporter signals while reducing assay costs by approximately 50 to 150-fold relative to commercial kits. Functional validation using the Keap1-Nrf2-ARE signaling pathway and carnosic acid demonstrated robust and expected reporter responses, with the N116H/FLUX^Vc^ system producing the strongest luminescence output. Furthermore, N116H enabled significantly improved real-time monitoring and single-cell imaging, producing up to 6.3-fold higher cumulative bioluminescence and increasing detectable cell numbers by approximately 4 to 5-fold compared with WT. Our findings establish N116H as a highly efficient and cost-effective luciferase variant for dual-reporter assays, real-time live-cell monitoring, and sensitive single-cell bioluminescence imaging applications.

Firefly luciferase (Fluc) is among the most widely used luciferases in reporter gene applications (1). It has been employed as bioreporters in cell-based assays and real-time monitoring applications due to its high sensitivity and rapid light emission (1, 2, 3, 4, 5, 6, 7). Fluc plays significant roles in drug screening and studying cellular functions, as it allows convenient assessment of promoter activities, which can be used to screen for activators or inhibitors as potential therapeutic agents (8, 9, 10). The advantages of Fluc in real-time monitoring and high-throughput screening (HTS) applications make it an ideal tool for screening natural product libraries (11) and accelerating the drug discovery process. Fluc can also be used for *in vivo* bioluminescence imaging to visualize and track cellular and molecular events in living organisms or experimental animals, such as in cancer research, for real-time monitoring of tumour progression and gene expression (12, 13, 14, 15). The ability of Fluc to generate sensitive and quantifiable luminescent signals is also instrumental for non-invasive imaging and real-time monitoring of other dynamic biological processes (16).

Fluc catalyzes the oxidation of D-luciferin (D-LH_2_) *via* reactions with adenine triphosphate (ATP) and oxygen with concomitant light emission (Fig. 1). First, a carboxyl group of D-LH_2_ is activated through adenylation by ATP, resulting in formation of an adenylated D-luciferin intermediate (**1.2**). The intermediate then reacts with oxygen to form a dioxetanone intermediate (**1.3a**) (17) and then proceeds to form the excited oxyluciferin (**1.4a**), the light emitting species, which then releases a photon while returning to the ground state oxyluciferin (**1.5**) as the final product. However, not all of the adenylated D-luciferin intermediate (**1.2**) goes through the light emitting path, as the reaction can also proceed through the path which forms hydrogen peroxide and adenylated dehydroluciferin (**1.3 b**), which can then bind tightly to Fluc with *K*_i_ of 3.8 ± 0.7 nM, inhibiting light generation (18). It was shown that species **1.3 b** can be converted to dehydroluciferin (**1.4 b**), a weaker inhibitor (*K*_i_ = 100–120 nM) (19) by addition of coenzyme A (CoA) or pyrophosphate. CoA reacts with **1.3 b** to form dehydroluciferyl-CoA (**1.5 b)** and then **1.4 b** by thiolysis, while pyrophosphate (PPi) can react with **1.3 b** *via* pyrophosphorolysis to form **1.4 b.** Although CoA is expensive, it serves as an effective light enhancing agent because it facilitates thiolysis of the adenylated dehydroluciferin inhibitor to generate a non-inhibitory CoA thioester and free Fluc for catalyzing new rounds of light-generating reactions. In contrast, an inexpensive PPi only facilitates the reverse adenylation equilibrium, which still allows the inhibitor to reform, leading to product inhibition and decreased signal stability. PPi also acts as a competitive inhibitor against ATP, resulting in reduced light signals. The addition of CoA is thus routinely used to enhance light signals of the Fluc reaction (20, 21, 22, 23, 24, 25, 26) by decreasing the enzyme species entrapped in the dark path that would otherwise lower light emission efficiency (27). Because CoA is expensive and unstable (28), the addition of CoA to increase and prolong bioluminescence signals (24, 25) adds extra cost to Fluc assays and drug screening programs (3).

**Figure 1:**
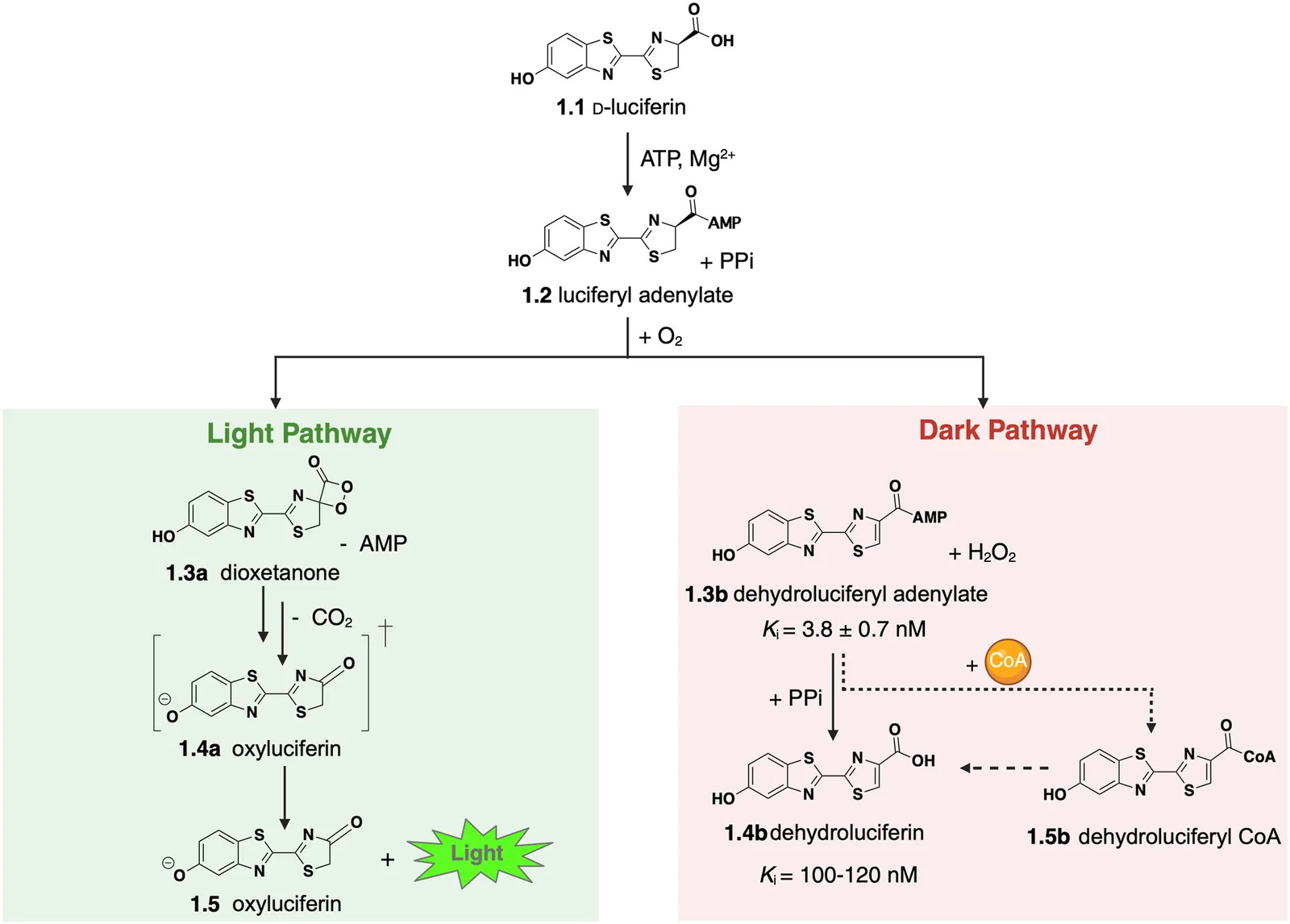
**Reaction mechanisms of firefly luciferase (Fluc).** Fluc catalyzes the oxidation of D-LH_2_ (1.1) with concomitant light emission *via* several steps. First, the carboxyl group of D-LH_2_ reacts with ATP to form the luciferin-adenylate intermediate (1.2). The luciferin-adenylate intermediate reacts with molecular oxygen to form the excited state oxyluciferin (1.4a) which emits a photon to form the final species, the ground state oxyluciferin (1.5). This reaction is proposed to occur *via* formation of a dioxetanone intermediate (1.3a) (17). However, a portion of the luciferyl-adenylate intermediate (1.2) can also undergo a side reaction, forming hydrogen peroxide and dehydroluciferyl adenylate (1.3b) which inhibits the luciferase reaction (*K*_i_ = 3.8 ± 0.7 nM) (18). This impacts the overall quantum yield of the bioluminescent reaction because part of the intermediate bifurcates into a dark pathway for each round of the catalytic cycle (27). Compound 1.3b can be converted to dehydroluciferin (1.4b), a weaker inhibitor (*K*_i_ = 100–120 nM) (19), through either pyrophosphorolysis or thiolysis with CoA. In pyrophospholysis, inorganic pyrophosphate (PPi) cleaves 1.3b to form 1.4b and ATP. In thiolysis, CoA reacts with the dehydroluciferin moiety of 1.3b to produce 1.5b, releasing AMP. Compound 1.5b can be further converted to 1.4b, allowing Fluc to be released and used in the following turnovers. In addition, the protonated oxyluciferin also inhibits luciferase (*K*_i_ = 500 ± 30 nM) (18).

Although Fluc from *Photinus pyralis* (*P. pyralis*) has been used in various applications mentioned above, the enzyme is not very stable and has a short half-life (*t*_1/2_) (29). Attempts in the past have been carried out to engineer Fluc to be more stable. Although many stable Flucs have been reported, their activities were mostly compromised and not very suitable for many bioreporter applications (30, 31, 32, 33). To the best of our knowledge, Fluc from *P. pyralis* developed by Promega Corporation is still the most widely used system (34, 35, 36). Therefore, a more thermostable Fluc with improved expression while still retaining the current functions would be beneficial for general applications of Fluc *in vitro* and *in vivo*.

Currently, several enzyme engineering approaches, particularly computational prediction, are available for improving enzyme stability with minimum perturbation of their activities (37, 38, 39, 40, 41, 42, 43). The semi-rational method, which combines the use of software predictions and site-saturation mutagenesis, is particularly useful for creating small and focused libraries with a high chance of obtaining enzymes with the desired properties (16, 39, 42, 44, 45). Recently, we found that mutations at surface residues of tryptophan monooxygenase resulted in variants with improved thermostability and soluble expression without compromising its catalytic activity (39).

In this work (Fig. 2), we engineered Fluc of *P. pyralis* using a semi-rational engineering approach to improve its thermostability and expression yield while maintaining bioluminescence activities. Two thermostable Fluc variants with enhanced light generation efficiency, N116H and N116H/M118L, were obtained. Bioluminescent properties were investigated using steady-state and pre-steady-state kinetics. The results revealed that these variants require substantially lower CoA supplementation, leading to the development of more economical Fluc assays. We constructed a novel cell-based dual-reporter Fluc and bacterial luciferase from *V.* *campbellii* (36) (Fluc/FLUX^Vc^) and developed in-house assay reagents. This system was designed to exploit the complementary advantages of two bioluminescent reporters, providing a robust and cost-effective platform for reporter gene assays and natural-product screening. Applications of the dual reporter system to measure the Keap1-Nrf2-ARE signaling pathway (46, 47, 48), real-time bioluminescence monitoring, and single-cell bioluminescence imaging were investigated. Real-time kinetic measurements in HepG2 cells expressing N116H revealed a stable 5-fold increase in signal brightness compared to the cells expressing the WT enzyme. Quantum yield (QY) measurement also confirmed a nearly 1.25-fold enhancement in the light emission efficiency of N116H compared to WT. Overall, this work resulted in the discovery of new Fluc variants and created a Fluc/FLUX^Vc^ dual-reporter platform as practical tools for sensitive and cost-effective bioluminescent systems for reporter gene analysis and screening applications.

**Figure 2:**
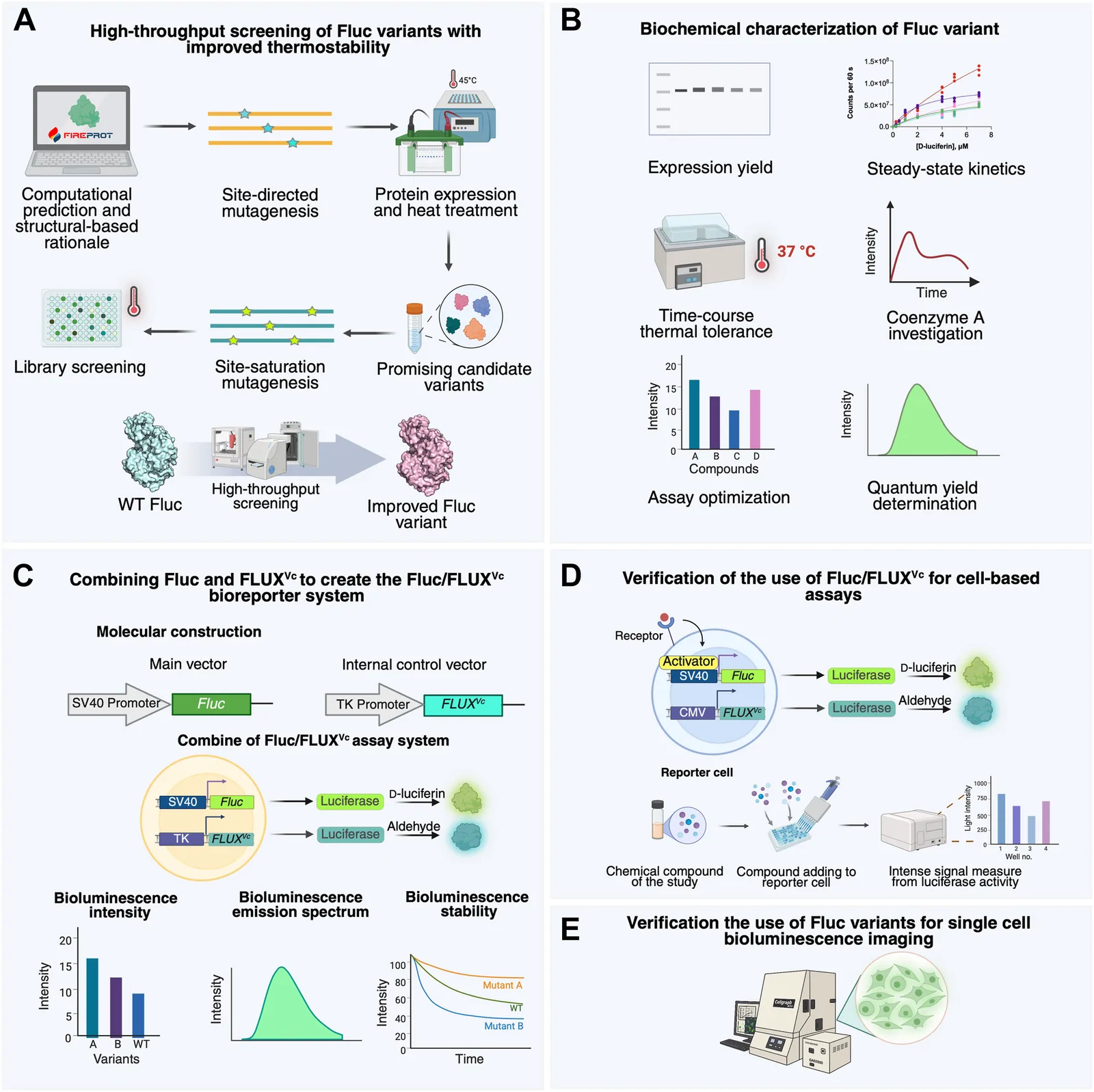
**Workflow of this study for engineering, investigating the catalytic properties and demonstrating applications of Fluc variants.***A*, semi-rational engineering was applied to identify Fluc variants with improved thermostability. Site-saturation mutagenesis at selected positions were used to generate variant libraries. *B*, biochemical and catalytic properties of Fluc variants were evaluated and compared to those of the WT enzyme. *C*, bioluminescence activities of Fluc variants expressed in cells were analyzed using a dual reporter combining the engineered Fluc/FLUX^Vc^ reporter system. *D*, application of the combined Fluc/FLUX^Vc^ reporter for cell-based assays. *E*, single cell bioluminescence imaging using the engineered Fluc variants.

## Results

### Engineering and selection of Fluc candidates with improved thermostability

To identify Fluc variants with improved thermostability, we employed a combined approach of using the FireProt program (49) to suggest promising candidates and performing site-saturation mutagenesis for creating variant libraries. This approach was recently employed to enhance the thermostability of tryptophan monooxygenase (TMO) with great success (39). Based on the crystal structure of Fluc (PDB ID: 4G36), the calculated folding energy and evolution conservation, FireProt predicted 36 single-point mutations which might result in enhanced thermostability (Table S1). We further rationally selected promising candidates using the structural selection criteria (39, 42). Residues within 15 Å of the active site were excluded to preserve the Fluc catalytic activity; surface mutations were preferably selected as they may promote stabilizing interactions such as salt bridges and hydrogen bonds and possibly increase soluble expression yields (50). These rationales eliminated 14 variants, leaving 22 candidates for experimental validation (Fig. 3 and Table S1). The constructed 20 variants (Table S1) were evaluated for their expression levels and thermal tolerance to heat treatment by SDS-PAGE analysis by and measuring the activities of crude lysates before and after incubating at 45 °C for 10 min.

**Figure 3:**
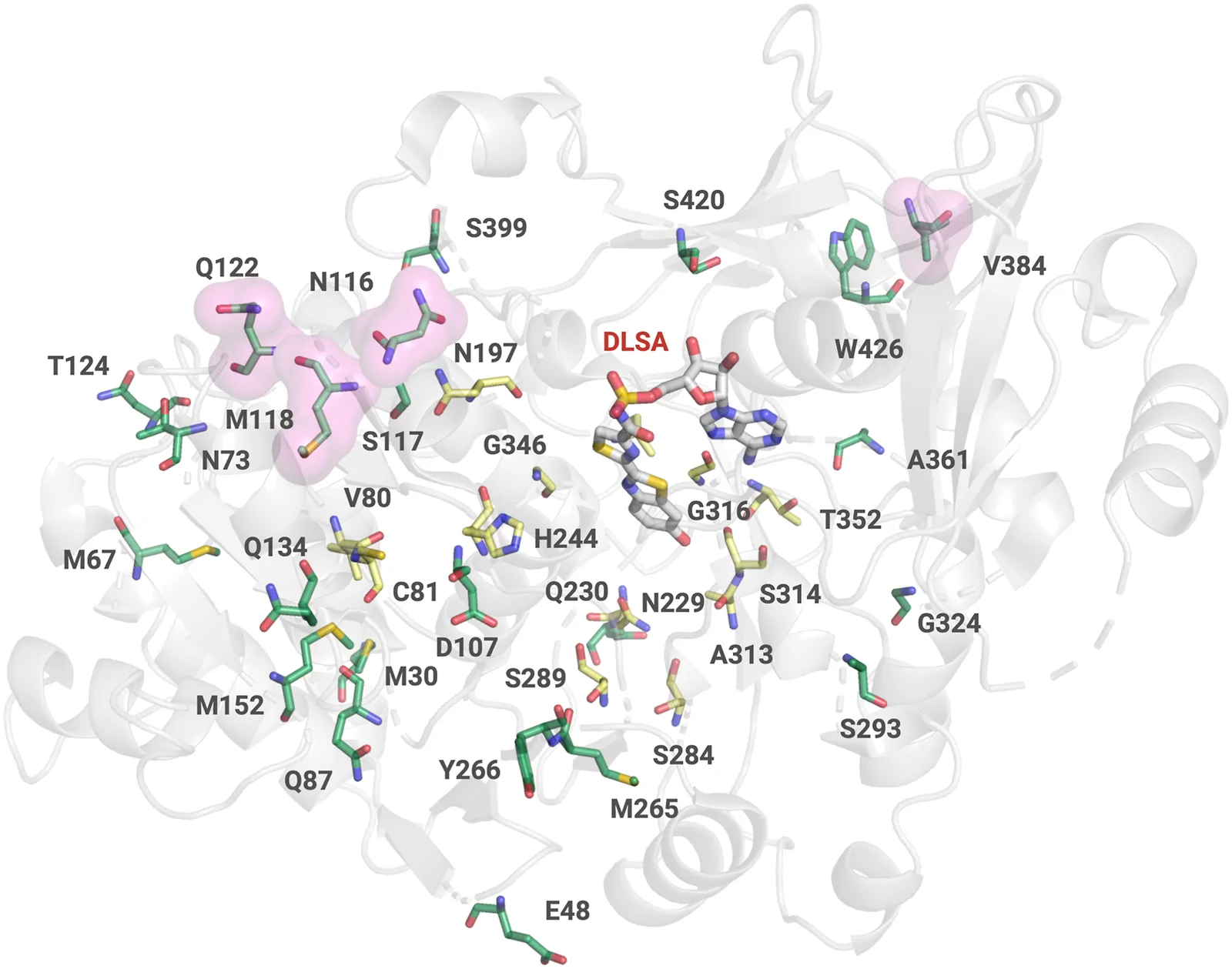
**Mutations suggested by the FireProt program** (49, 79, 80)**.** Locations of 36 mutations suggested by the FireProt program are shown by displaying sidechains (*sticks*) for the residues using PyMOL 2.5 (Schrödinger) (https://pymol.org). Surface mutations are shown in *green* and internal mutations in *yellow*. Residues within 15 Å to the active site were excluded to preserve catalytic activity, resulting in 22 mutations around the surface area. Residues identified by our initial screening (N116, M118, Q122, and V384) were further subjected to site-saturation mutagenesis and are highlighted with a *pink* surface. Atoms are colored according to the standard color codes with oxygen in *red*, nitrogen in *blue*, and sulfur in *light orange*.

We found that only lysates of seven variants (N73K, N116H, M118L, Q122K, Q134P, V384P, and S399K) retained measurable bioluminescence activities after the heat treatment (Fig. S1). All of these seven variants were then purified and further analyzed for their activities. The results showed that only four of these variants—N116H, M118L, Q122K, and V384P—exhibited high soluble expression levels. Therefore, these four positions were selected for further site-saturation mutagenesis to comprehensively screen for variants with the best thermostability and enhanced soluble expression.

### Identification of variants with improved thermostability *via* site-saturation mutagenesis at N116, M118, Q122, and V384 positions

Guided by prior FireProt analysis and site-directed mutagenesis results, we performed site-saturation mutagenesis at the N116, M118, Q122, and V384 positions using degenerate NNK primers (Table S2) to construct saturation mutagenesis libraries encompassing all 20 amino acid substitutions at each target position. More than 2800 clones were screened using the workflow shown in Figure S2 to identify variants with enhanced residual activities following thermal challenge at 45 °C for 30 min. We identified 16 variants exhibiting 4- to 20-fold higher activity than WT (Fig. S3). Sequencing results revealed the substitutions with positive results to be N116 (A, H, V, W; ∼7-fold increase), M118 (V, L, R; ∼20-fold), Q122 (S, A, E, R, L; ∼4-fold), and V384 (D, T, L, G; ∼7-fold). As lysate activities during the screening process could vary significantly, we thus partially purified Fluc variants using poly(ethyleneimine) (PEI) and ammonium sulfate precipitations and applied heat treatment at 45 °C for 20 min to the crude lysates. The results showed that N116H and M118L displayed significantly higher activities than WT, while Q122A and V384D showed activities comparable to WT (Fig. S4).

### Steady-state kinetics of light emission and expression yields identified Fluc N116H, M118L, and N116H/M118L variants as promising candidates

Catalytic properties of purified top-performing variants (N116H, M118L, Q122A, and V384D) were investigated using steady-state kinetics performed at 25 °C and pH 8.0, in comparison to WT (Experimental procedures, Table 1 and Fig. S5). The results showed that Q122A and V384D exhibited substantially lower *K*_m_ values for D-LH_2_ of 1.10 μM and 0.75 μM, respectively, compared to 3.64 μM for WT. However, this decrease in *K*_m_ was accompanied by a pronounced decrease in *k*_cat_, as reflected by approximately10-fold lower *k*_cat_ values and reduced *V*_max_ in the 10^5^ count per sec (CPS) range. In contrast, N116H, M118L, and the double variant N116H/M118L maintained similar *K*_m_ values to the WT enzyme (2.49, 2.28, and 3.73 μM for N116H, M118L and N116H/M118L, respectively) while exhibiting markedly enhanced *k*_cat_ values. Among these, N116H/M118L showed the greatest improvement, reaching a *V*_max_ of 8.51 × 10^6^ CPS and 5.3-fold increase in relative *k*_cat_. N116H and M118L also showed substantial increases in activity, with 4.6-fold (7.40 × 10^6^ CPS) and 3.5-fold (5.65 × 10^6^ CPS) enhancement, respectively. The increased *k*_cat_ values or enhanced light output (photons emitted per second per enzyme molecule) of N116H, M118L, and N116H/M118L make these variants promising candidates for improving the sensitivity of Fluc assays. By maintaining *K*_m_ while enhancing *k*_cat_, these variants significantly amplified their overall catalytic efficiency (*k*_cat_/*K*_m_) compared to WT. This higher *k*_cat_ possibly stems from the surface mutation changing the global conformational dynamics, which can thereby support the catalytic activity by lowering the energetic barriers for the rate-limiting steps, facilitating more efficient product release and preventing the enzyme from entering non-productive states (17, 23).

**Table 1:** Kinetic parameters, expression yield, and times required for the enzyme to display 50% of its original activity (half-lives, *t*_1/2_) at 37 °C of Fluc variants and WT

Enzyme	^app^*K*_m_, μM	*V*_max_, CPS (x 10^6^)	Relative *k*_cat_	Relative *k*_cat_ (fold-change)	Expression yield (mg purified Fluc/L culture)	Expression yield (fold-change)	^37^*t*_1/2_, min
WT	3.64 ± 0.18	1.60 ± 0.09	100.0	1.0	17.4	1.0	13
N116H	2.49 ± 0.12	7.40 ± 0.44	461.5	4.6	47.1	2.7	46
M118L	2.28 ± 0.14	5.65 ± 0.28	352.3	3.5	65.1	3.7	21
N116H/M118L	3.73 ± 0.26	8.51 ± 0.60	531.0	5.3	69.2	4.0	37

Consistent with the observed steady-state kinetic enhancements, N116H, M118L, and N116H/M118L exhibited substantially improved soluble expression yields compared to the WT enzyme (Table 1 and Figs. S5 and S6). While a 1 L culture of the WT enzyme yielded 17.4 mg of purified enzyme, a 1 L culture of N116H, M118L and N116H/M118L yielded 47.1 mg, 65.1 mg and 69.2 mg/L of the corresponding purified enzymes, respectively. These results indicate that the soluble expression yields of N116H, M118L, and N116H/M118L were around 3- to 4-fold greater than WT.

Soluble expression yield, *k*_cat_, and thermal stability (*T*_m_ and half-life) were positively correlated. These surface mutations improved both physical and catalytic properties at the same time. Making the surface more rigid may help the protein fold properly, driving the 3- to 4-fold yield increase, while also keeping the enzyme stable during reactions. To further confirm the selection of these variants using purified enzymes, we measured luciferase activities of all four top-performing variants after heat treatment at 45 °C for 20 min N116H and M118L showed significantly higher activity than the WT enzyme (3.5- and 1.6-fold, respectively), whereas Q122A and V384D showed significantly lower activity than the WT enzyme (Fig. S7). Based on the results in Table 1 and Figures S5–S7, Q122A and V384D were excluded from further analysis. Only the N116H, M118L, and N116H/M118L (combining double mutations of N116H and M118L) variants were chosen for further investigation of their biochemical and biophysical properties.

### Thermostability of Fluc variants

To further evaluate the thermal stability of N116H, M118L and N116H/M118L variants compared to WT, their *T*_m_ values were determined using thermal shift assays (Experimental Procedures). The results showed that *T*_m_ values of WT (36.0 ± 0.0 °C), N116H (38.0 ± 0.0 °C), M118L (35.7 ± 0.6 °C), and N116H/M118L (37.0 ± 0.0 °C) were all in the same range (Fig. S8, *A* and *B*). We subsequently measured their residual luciferase activities during prolonged incubation at 37 °C, the condition normally used for luciferase assays. While the WT enzyme demonstrated a time required to reduce the activity to 50% of the original value (half-life, *t*_1/2_) of 13 ± 1.0 min, all variants N116H, M118L, and N116H/M118L exhibited significantly longer *t*_1/2_ values of 46 ± 2.9 min, 21 ± 1.2 min, and 37 ± 6.7 min, respectively (Table 1. and Fig. S8, *C* and *D*). The data indicate that these variants were more stable than WT. This clear contrast—where *t*_1/2_ increases dramatically despite nearly identical *T*_m_—shows that the surface mutations slow down inactivation rather than changing the overall protein melting balance (51, 52). Although the *T*_m_ values stayed within a narrow range (35.7–38.0 °C), the modifications drastically extended how long the variants stay active at 37 °C. This implies that the surface mutations selectively block the initial, local unfolding steps, keeping the enzyme working longer without shifting its stability profile (51, 52). Since M118L showed the shortest *t*_1/2_ among all variants, it was excluded from further investigation. Therefore, only the N116H and N116H/M118L variants were further investigated for their biochemical, biophysical and bioluminescence properties.

### Evaluation of additive effects (EDTA, DTT, and BSA) on WT, N116H, and N116H/M118L variants

In addition to engineering Fluc to improve its functions, we investigated the influence of several additives—ethylenediaminetetraacetic acid (EDTA), dithiothreitol (DTT), and bovine serum albumin (BSA)—on the bioluminescence intensity of WT, N116H, and N116H/M118L.

First, the effects of EDTA, a metal chelator that sequesters divalent metal ions, were examined. Activities of WT and the N116H and N116H/M118L variants were the highest in the absence of EDTA. At concentrations of 0.625 to 5 mM EDTA, their activities decreased, and they were particularly low at 10 mM EDTA (Fig. S9, *A*–*C*). These effects are probably caused by chelation of Mg^2+^ by EDTA. Mg^2+^–ATP is indispensable and required for the formation of the luciferyl adenylate intermediate (Fig. 1, 1.2).

Next, the effect of DTT, a reducing agent that maintains cysteine residues in a reduced state, was investigated. Under our assay conditions, Fluc WT activities were similar between 0 and 2.5 mM DTT, but their values were significantly lower at higher concentrations of DTT (Fig. S9*D*). For the N116H and N116H/M118L variants, the addition of DTT decreased enzyme activities (Fig. S9, *E*–*F*). Our results differ from previous reports describing enhancement of bioluminescence after 1 h incubation of WT with 0.25 to 1 mM DTT (53).

Additionally, the effect of BSA, a known surface-blocking and stabilizing agent reported to enhance bioluminescence intensity at 0.6 to 1.25 mg/ml (53), was examined. Bioluminescence enhancement was not observed in our systems (Fig. S9, *G*–*I*). Generally, the presence of BSA resulted in comparable or slightly decreased bioluminescence signals for all tested Fluc reactions.

Overall, these results indicated that EDTA, DTT, and BSA did not enhance bioluminescence intensity in any Fluc enzymes under the conditions developed in this work. Therefore, these additives were omitted from the final assay formulation.

### Investigation of coenzyme A effects on Fluc variants

Fluc uses D-LH_2_, ATP, and Mg^2+^ as substrates to generate a flash burst of light followed by a slow light decay. The initial flash burst is generated from the first turnover reaction of Fluc. During subsequent turnovers, Fluc reactions increasingly accumulate the dehydroluciferyl-adenylate intermediate (Fig. 1; 1.3b). As the intermediate 1.3 b tightly binds to the enzyme's active site and the complex does not generate light, subsequent turnover reactions result in decreased light emission (23, 54). Addition of CoA helps increase the steady-state light signals of the following turnovers by promoting thiolysis of dehydroluciferyl-adenylate (Fig. 1; 1.3b) to form dehydroluciferyl-CoA (Fig. 1; 1.5b), which can dissociate from Fluc to liberate the active enzyme for generating bioluminescence in the following reactions and increasing light output (23).

To investigate CoA effects on bioluminescence, the reactions of purified WT, N116H, and N116H/M118L were assayed in the presence of varying CoA concentrations (0–3.2 mM). The results showed that CoA enhanced the light output for all Fluc reactions but to different degrees (Fig. 4, *A*–*C*). The bioluminescence of WT was strongly dependent on CoA (0–2.4 mM), with a maximum intensity at 2.4 mM CoA (Fig. 4*A*). In contrast, bioluminescence intensities of N116H and N116H/M118L (Fig. 4, *B* and *C*) exhibited substantially reduced dependence on CoA, and only CoA at 0.16 to 2.4 mM increased the light intensity modestly. Nonetheless, at 3.2 mM CoA, activities of all enzymes diminished significantly (Fig. 4, *A*–*C*), suggesting that excess CoA exerts inhibitory effects.

**Figure 4:**
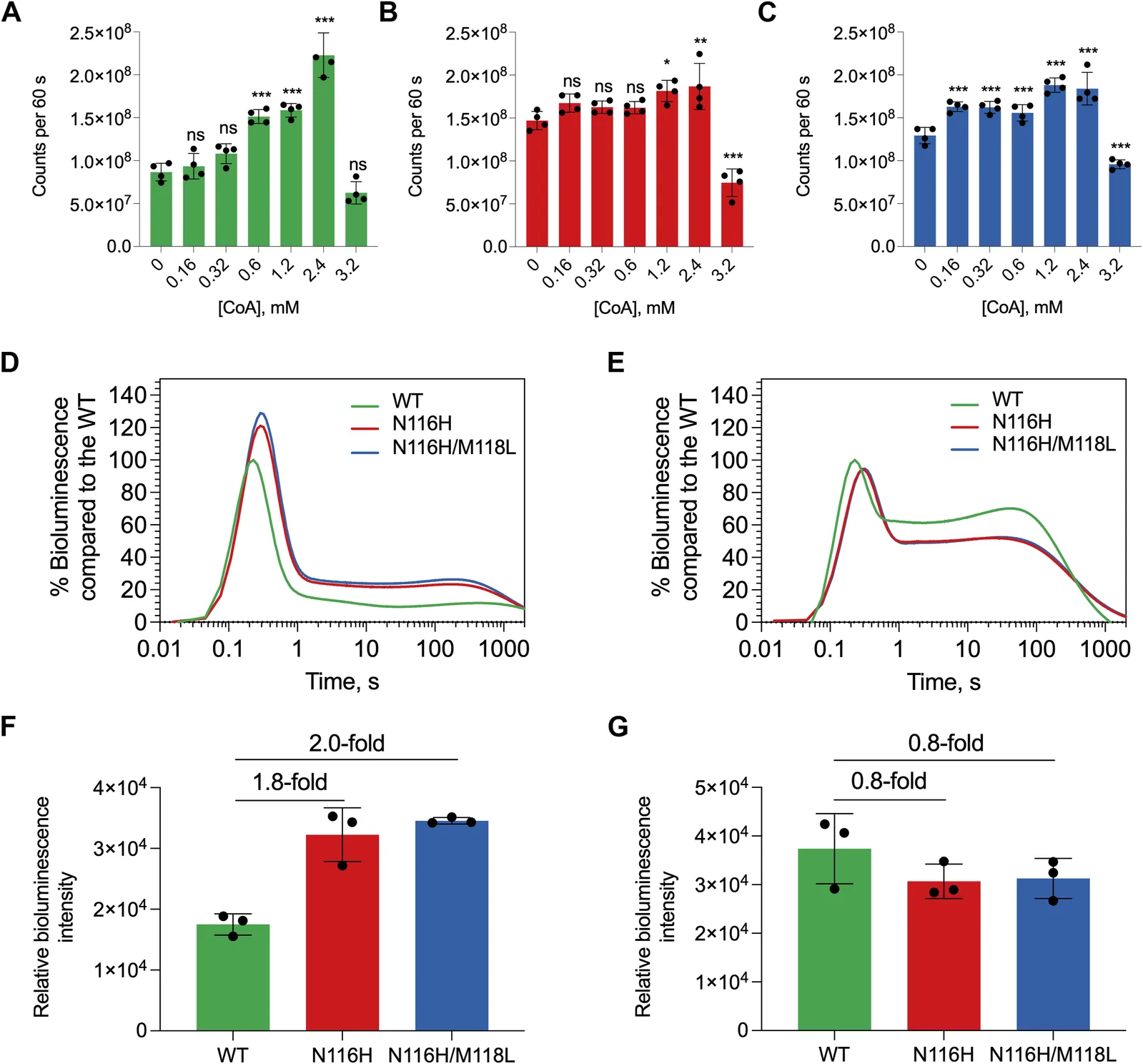
**Effects of coenzyme A (CoA) on Fluc.***A–C*, activities of purified Fluc (*A*) WT, (*B*) N116H, and (*C*) N116H/M118L variants. Each purified variant (1 μM) was pre-mixed with CoA at varying concentrations and incubated for 10 min before activity measurements. Data are presented as mean ± S.D. (*n* = 4). One-way ANOVA was used for statistical analysis; ∗*p* < 0.01, ∗∗*p* < 0.001, ∗∗∗*p* < 0.0001; ns = not significant. *D* and *E*, bioluminescence kinetics of WT (*green*), N116H (*red*), and N116H/M118L (*blue*) variants in the absence (*D*) or presence (*E*) of 0.1 mM CoA. Reactions were initiated by rapidly mixing luciferase (0.25 μM final) with substrates (40 μM D-LH_2_, 3 mM ATP, 6 mM MgCl_2_ final) in 100 mM Tris-HCl (pH 8.0) using a stopped-flow spectrometer in light-off mode. Kinetics of bioluminescence with λ_em_ ≥ 325 nm at 25 °C were monitored for 2000 s. *F* and *G*, comparisons of bioluminescence intensity calculated from the area under the curve (AUC) from the bioluminescence kinetics. *F*, in the absence or (*G*) presence of 0.1 mM CoA. Data represent mean ± S.D. (*n* = 3).

We further determined the limit of detection (LOD), or the lowest amount of Fluc that gives reliable detection of WT and variants, at varying CoA concentrations (Fig. S10). In the absence of CoA, both N116H and N116H/M118L variants exhibited superior sensitivity because their LOD values (0.05 nM and 0.09 nM for N116H and N116H/M118L, respectively) are lower than that of WT (0.13 nM). Upon the addition of 0.1 mM CoA, LOD values became comparable among all enzymes (0.04, 0.07, and 0.07 nM for WT, N116H and N116H/M118L, respectively).

All these results suggested that for bioluminescence assays using WT Fluc, addition of CoA is necessary to enhance the overall bioluminescence, consistent with previously reported results (19, 21, 23, 54, 55). However, the assays using the N116H and N116H/M118L variants do not require CoA because the reactions without addition of CoA or adding CoA at 0.1 mM remained capable of generating strong bioluminescence signals.

### Transient kinetics to investigate the effects of CoA on Fluc reactions

To gain mechanistic insights into CoA-dependent kinetics, we employed stopped-flow techniques to monitor pre-steady-state and steady-state bioluminescence of WT and the N116H and N116H/M118L variants. Consistent with the previous findings (23, 54, 55), the reaction of WT Fluc in the absence of CoA displayed rapid light generation from 0.002 s to 0.229 s, followed by light decay from 0.229 s to 0.993 s during the first turnover reaction. After approximately 1.02 s, the reaction entered the steady-state phase of light generation (Fig. 4*D*). Addition of 0.1 mM CoA substantially increased steady-state light output, and we noted that the reaction of WT exhibited a pronounced second phase of light generation and decay upon CoA addition (Fig. 4*E*). These results clearly indicate a rescue of the inhibited enzyme population.

For the reactions of N116H and N116H/M118L variants in the absence of CoA, the level of light generated during the first turnover and following steady-state reactions were higher than that in the reaction of WT. The kinetic behavior suggests that accumulation of the dehydroluciferyl-adenylate intermediate in these variants is likely less than that found for WT. This is consistent with the smaller enhancement by CoA on their reactions (Fig. 5). The results indicate the reactions of N116H and N116H/M118L bifurcate less to the dark path than does the WT reaction, resulting in less dependency on CoA to liberate free enzymes from the dead-end inhibited complex.

**Figure 5:**
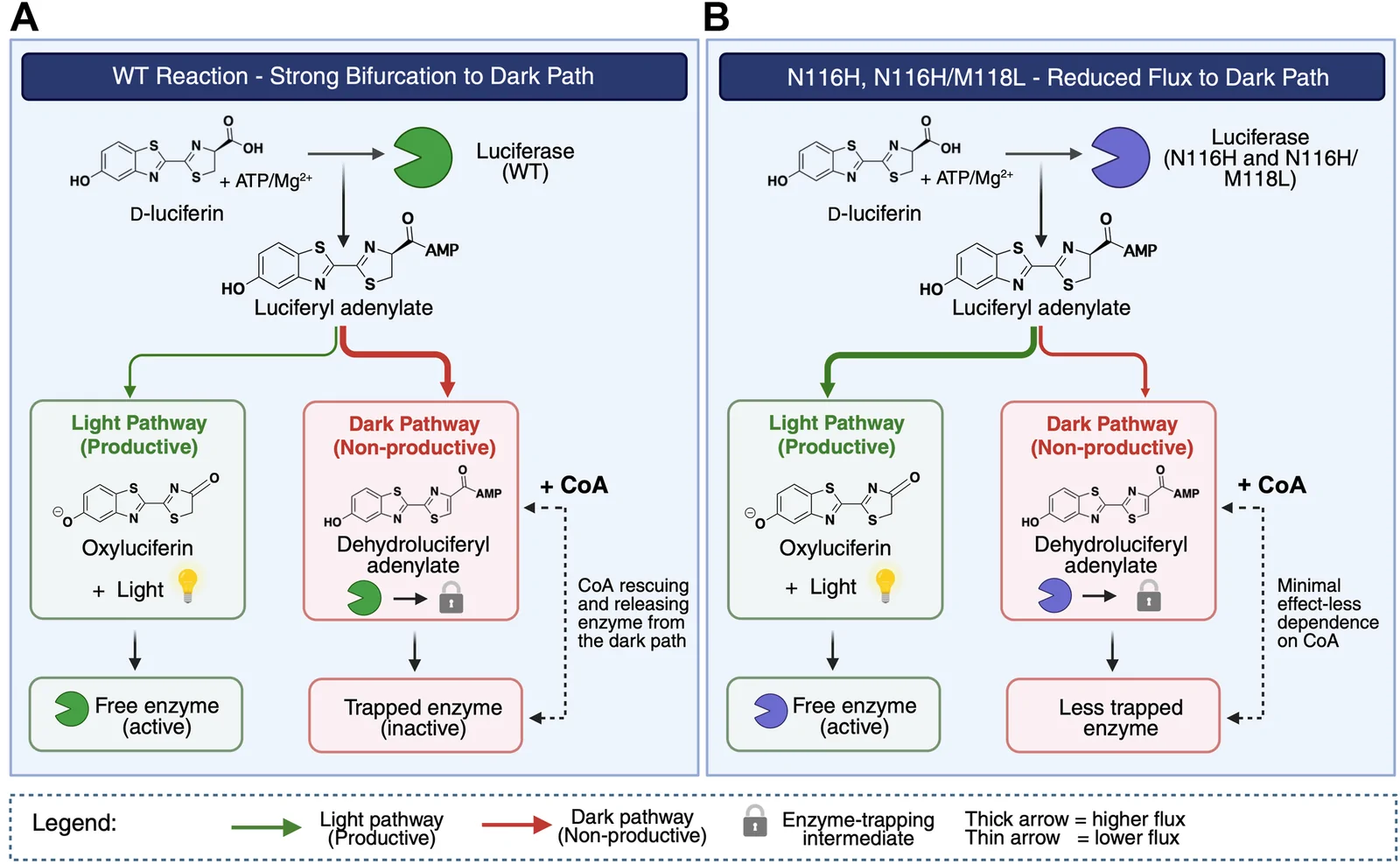
**Model explaining the enhanced bioluminescence light output in Fluc variants.** Schematic diagram of reaction pathways for (*A*) WT and (*B*) Fluc variants (N116H and N116H/M118L) in the absence or presence of CoA. WT bifurcates to a non-productive pathway that forms a dehydroluciferyl adenylate intermediate and traps the enzyme more than the Fluc variants.

Quantitative analysis of bioluminescence intensity in the absence of CoA and 0.1 mM CoA confirmed the reduced CoA dependency for Fluc variants (Fig. 4, *D* and *E*). In the absence of CoA, both variants produced higher light intensities than those of the WT enzyme (Fig. 4, *D* and *F*). However, in the presence of CoA, the bioluminescence intensity of WT was significantly enhanced compared with the variants (Fig. 4, *E* and *G*). The results indicate that bioluminescence of all of the enzymes can be enhanced by the addition of CoA, with the WT Fluc showing stronger CoA dependency than the N116H and N116H/M118L variants. The data suggest that bioreporter applications using these Fluc variants do not require CoA addition and would save the overall assay cost significantly compared to the use of WT Fluc.

### Construction of a cost-effective dual-reporter system, measurement of cellular activities of Fluc variants and its validation as a bioreporter

A bioreporter assay needs to generate a measurable signal in response to specific stimuli, providing a rapid and sensitive platform for biological evaluation (56, 57, 58). For a luciferase-bioreporter requiring expression in cells, a system normally consists of a target reporter that reports signals according to stimuli and another system that is an internal control reporter independent of stimuli for generating signals to normalize transfection efficiency and cell numbers. A dual-reporter widely used is a combination of Fluc as the target reporter and Rluc as the internal control reporter for normalization. However, as coelenterazine, a substrate for Rluc is relatively expensive, here we constructed a cost-effective reporter that can reduce the cost of cell-based bioluminescence assays significantly by combining Fluc variants identified in this study and a bacterial luciferase from *V. campbellii* (FLUX^Vc^) as an alternative internal control reporter (36). As the engineered Fluc does not require CoA for their reactions (Figs. 4 and 5), we thus developed in-house assay reagents omitting CoA to further reduce the cost for the Fluc/FLUX^Vc^ dual reporter (Table S3, Fig. S12). By comparing to the commercially available assay reagents, our formulated in-house reagents for the Fluc/FLUX^Vc^ dual reporter assays could reduce the cost from $2.10 or $6.45 per assay to $0.043, accounting for 50- to 150- fold cost reduction (Table S4).

To establish a robust and economical bioreporter platform, we thus evaluated the use of Fluc/FLUX^Vc^ dual reporter of N116H and N116H/M118L in comparison to WT Fluc and to the results obtained from the standard Fluc/Rluc system (Fig. 6) across three cell lines: HEK293T, HepG2, and HeLa cells. These cells were chosen because HEK293T cells provide high transfection efficiency and strong signal output (59, 60, 61), HepG2 cells offer a liver-like metabolic environment suitable for drug-related studies (61, 62), and HeLa cells are highly proliferative and easy to transfect (61, 63). They are widely used for reporter-based assays and comparative expression studies.

**Figure 6:**
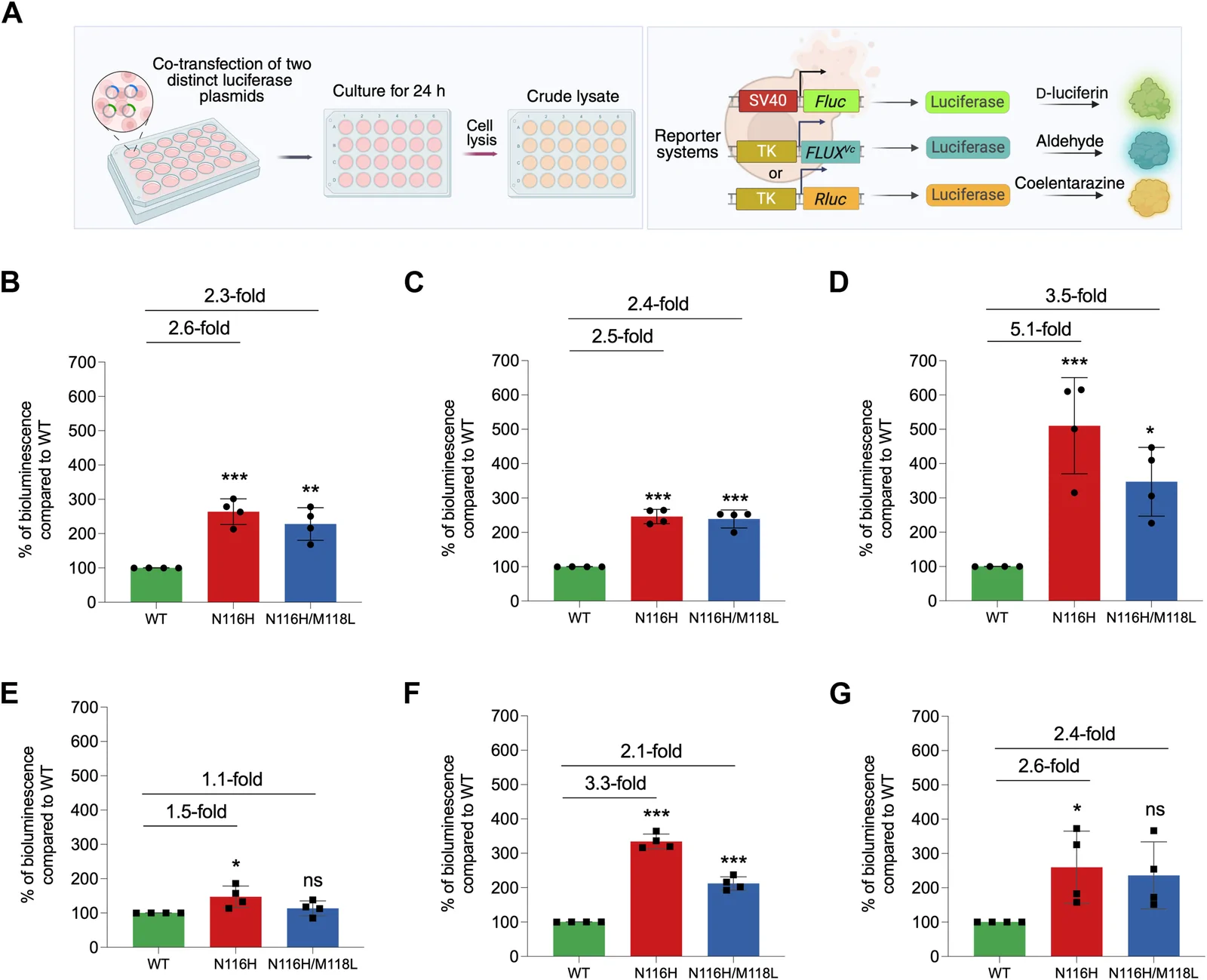
**Comparison of bioluminescent signals from different cell lines carrying WT, N116H, and N116H/M118L reporter systems.***A*, crude lysates from cell-line expressing different luciferases were evaluated using two assay systems. Fluc activity assays were used as the main system with either bacterial luciferase (FLUX^Vc^) or renilla luciferase (Rluc) assays as an internal control. *B–D*, results from using FLUX^Vc^ (•) while (*E–G*) were results from using Renilla luciferase (Rluc) (▪). Cell lines were (*B, E*) HEK293T, (*C, F*) HepG2, and (*D, G*) HeLa cells. Fluc activities were normalized based on the internal control signals and expressed as % of bioluminescence compared to WT. Data represent mean ± SD (*n* = 4). Statistical significance was determined by one-way ANOVA: ∗*p* < 0.01; ∗∗*p* < 0.01; ∗∗∗*p* < 0.001; ns = not significant.

We initially evaluated the performance of engineered Fluc/FLUX^Vc^ dual reporter using HEK293T cells. HEK293T cells were transiently co-transfected with pGL4-SV40-*Fluc* (WT or variants) and pGL3-TK*-FLUX*^*Vc*^, where their expressions were regulated under the SV40 and TK promoter, respectively (Figs. S11, *A* and *B* and S12). After co-transfection of these plasmids, cells were lysed using 200 μl of in-house lysis buffer (Table S3) or Passive Lysis Buffer (5x; Promega, Cat. No. E1941). This volume was previously optimized to ensure pipetting accuracy for cell lysis (Fig. S12*A*). Bioluminescence signals obtained from the two lysis buffers were comparable (Fig. S12, *B* and *C*), indicating similar cell lysis efficiency by commercially available and in-house lysis buffers. Remarkably, N116H exhibited the highest bioluminescence intensity, 2.8- or 2.7-fold higher than that of the WT when using either Passive lysis buffer (Promega) or in-house lysis buffer, respectively (Fig. S12, *B* and *C*). N116H/M118L also showed greater bioluminescence intensity, 1.6- or 1.8-fold when using Passive lysis buffer or in-house lysis buffer, respectively.

We then compared the performances of the engineered Fluc/FLUX^Vc^ with the known Fluc/Rluc dual bioreporter across cell lines—HEK293T, HepG2, and HeLa cells. Fluc expressed in each dual bioreporter system was either WT or variants (Figs. 6 and S13). Therefore, each cell line was co-transfected with pGL4-SV40-*Fluc* (WT or variants) and either pGL3-TK*-FLUX*^*Vc*^ or pRL-TK*-Rluc*. After 24 h, cells were lysed, and the activity was measured using D-LH_2,_ aldehyde, or coelenterazine, according to their substrates (Fig. 6*A*) as described in Experiment Procedures. Notably, both N116H and N116H/M118L exhibited greater bioluminescence than the WT; the signals from N116H were the highest across all three cell lines (Figs. 6 and S13). HEK293T expressing N116H and N116H/M118L exhibited 2.6-fold and 2.3-fold greater bioluminescence than the WT, respectively (Figs. 6*B* and S13*A*). Similar enhanced bioluminescence levels were observed for HepG2 expressing these variants (Figs. 6*C* and S13*B*) while HeLa cells expressing N116H and N116H/M118L showed 5.1-fold and 3.5-fold increase over WT, respectively (Figs. 6*D* and S13*C*).

Similar bioluminescence signals were also observed between the Fluc/FLUX^Vc^ and Fluc/Rluc systems (Figs. 6 and S13), confirming that FLUX^Vc^ can serve as a reliable internal control reporter. When Rluc was used as the internal control reporter (Fig. 6, *E*–*G*), N116H showed the highest bioluminescence in HepG2 and HeLa cells, with 3.3-fold and 2.6-fold greater than the WT (Fig. 6, *F* and *G*). However, only a modest increase (∼1.5-fold) was observed in HEK293T cells (Fig. 6*E*).

Analysis of raw data values showed that HEK293T cells produced the highest absolute reporter activity among all three cell lines (Fig. S13). FLUX^Vc^-normalized signals were comparable across cell types (Fig. S13, *D*–*F*), whereas Rluc activity was markedly higher in HEK293T cells (Fig. S13*J*). This may be due to the higher plasmid uptake and stronger transcriptional capacity for HEK293T (64, 65). Despite their lower absolute signals, HeLa cells exhibited the greatest fold change for both variants (Fig. S13, *C* and *I*), because normalization to a similar internal control across cell lines reveals a larger relative induction in HeLa cells.

Finally, emission spectra of HepG2 expressing WT and mutants were characterized. While N116H and N116H/M118L produced higher bioluminescence intensities, their emission maxima derived from purified enzymes (Fig. 7) and living cells (Fig. S14) were identical to that of WT, indicating that the mutations enhanced catalytic efficiency without altering emission properties.

**Figure 7:**
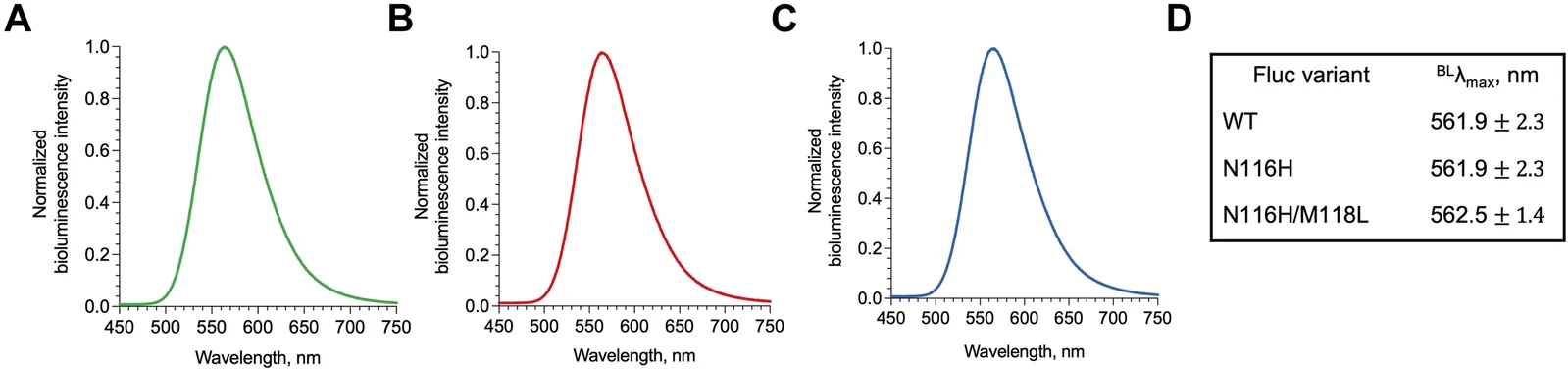
**Bioluminescent emission spectra of the purified Fluc variants.***A*, WT (*green*), (*B*) N116H (*red*), and (*C*) N116H/M118L (*blue*). Data are presented as an average from three replicates. *D*, Emission wavelengths of WT, N116H and N116H/M118L.

All results suggested that N116H and N116H/M118L are expected to improve assay sensitivity and dynamic range in HTS applications due to their stronger bioluminescence output than WT. N116H particularly exhibited the strongest bioluminescence across all cell lines (Fig. 6, *B*–*G*, S13, *A*–*C* and *G*–*I*), with up to a 5.1-fold increase in HeLa cells using FLUX^Vc^ for normalization (Fig. 6*D*). The increased bioluminescence observed is consistent with its higher *k*_cat_ value (Table 1 and Fig. S5).

### Verification of the Fluc/FLUX^Vc^ dual-reporter system in cell-based assays

To demonstrate the applicability of the dual Fluc/FLUX^Vc^ reporter system for cell-based assay analysis, the Keap1-Nrf2-ARE pathway, a key regulator of cellular oxidative stress responses (46, 47, 48, 66), was used as a model for testing. Carnosic acid, a well-characterized Nrf2 activator that can modify Keap1’s cysteine residues (67, 68), was used as a model Nrf2 activator for this study.

A dual-reporter system was constructed in which the antioxidant response element (ARE) cassette driving Fluc expression was inserted into a linearized pGL4 plasmid (Fig. S11*C*). In parallel, the FLUX^Vc^ plasmid under the control of a constitutive CMV promoter was constructed in a pcDNA3 plasmid and used as an internal control (Fig. S11*D*). HepG2 cells were co-transfected with pcDNA3-*FLUX*^*Vc*^ and pGL4-ARE-minP-*Fluc* (WT, N116H, or N116H/M118L). After 24 h, cells were treated with increasing concentrations of carnosic acid (0–100 μM) for an additional 24 h, followed by cell lysis and bioluminescence measurement using D-LH_2_ (Fig. 8*A*).

**Figure 8:**
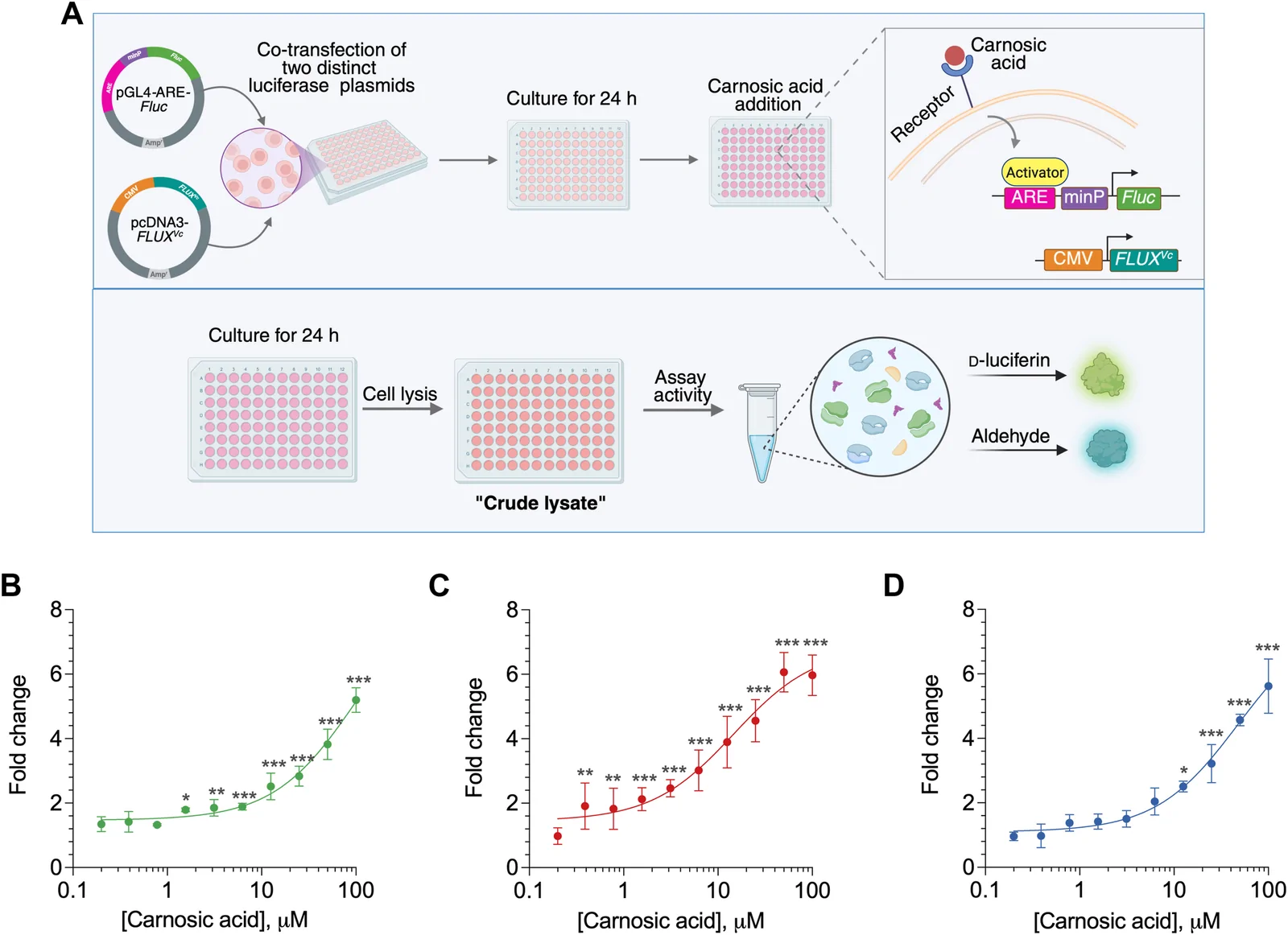
**Nrf2 activation by carnosic acid in HepG2 cells measured using the dual luciferase reporter system consisting of Fluc variants and bacterial luciferase.***A*, HepG2 cells were seeded in 96-well plates and co-transfected with 200 ng of a Fluc reporter plasmid—pGL4-ARE-minP*-Fluc*-WT (*green*), N116H (*red*), or N116H/M118L (*blue*)—and 20 ng of the FLUX^Vc^ internal control plasmid (pcDNA3-*FLUX*^*Vc*^). After 24 h, cells were treated with a two-fold dilution of carnosic acid. *Panels* (*B–D*) show fold-change values normalized to FLUX^Vc^ and DMSO (0.1%). Dose–response curves were fitted by nonlinear regression in *GraphPad Prism* 10.6.1. Data represent mean ± SD (*n* = 3), and statistical significance was determined by one-way ANOVA: *p* ∗< 0.01; ∗∗*p* < 0.01; ∗∗∗*p* < 0.001.

Treatment with carnosic acid resulted in a dose-dependent increase in Fluc activity across all variants (Fig. 8, *B*–*D*). The results were consistent with those previously reported (67, 68, 69, 70), suggesting that increasing carnosic acid progressively enhances ARE-driven transcription, promoting elevated bioluminescence signals as expected. Among the tested variants, N116H showed significantly higher bioluminescence intensity compared to WT and N116H/M118L, particularly at ≥ 3.3 μM carnosic acid (Fig. 8*C*), suggesting enhanced reporter responsiveness to Nrf2 activation. For N116H/M118L (Fig. 8*D*), the bioluminescence signals were comparable to WT across all treatment conditions (Fig. 8*B*). Overall, N116H showed a greater response to carnosic acid in the Keap1-Nrf2-ARE pathway than WT and N116H/M118L. However, the basis for this enhanced responsiveness remains unclear and requires further investigation.

All data also support that the dual Fluc/FLUX^Vc^ reporter serves as a reliable cellular bioreporter because the observed increase in Fluc activities was specific through the activation of the Keap1-Nrf2-ARE pathway rather than variations in transfection efficiency or cell viability. Carnosic acid did not affect the internal control reporter as the FLUX^Vc^ signals remained stable across all treatment conditions (Fig. S15). This signal stability validates the suitability of using FLUX^Vc^ as an internal control reporter for signal normalization. Altogether, the developed Fluc-N116H/FLUX^Vc^ dual bioreporter system is robust and applicable to screening and evaluation of drugs and natural products.

### Evaluation of Fluc variants for non-disruptive real-time monitoring and single-cell bioluminescence imaging

To evaluate Fluc performance in real-time bioluminescence monitoring, WT and engineered variants (N116H and N116H/M118L) were cloned into the pGL4 luciferase reporter vector under the SV40 promoter and expressed in HepG2 cells. Total bioluminescence with 10 s light integration was recorded every 30 min for a total period of 72 h. The results showed that the different variants displayed distinct bioluminescence kinetic profiles (Fig. S16, *A*–*C*). Although WT displayed lower intensity throughout the monitoring period, its light decayed at a slower rate (Fig. S16*A*). A gradual signal reduction was observed for all variants between 48 and 72 h (Fig. S16, *A*–*C*). The area under the curve (AUC) values were ranked as WT < N116H/M118L < N116H, with cumulative light output increasing by 6.3-fold and 5.0-fold for N116H and N116H/M118L, respectively (Fig. S16*D* and Table S5).

We further assessed the detection sensitivity of the engineered variants and WT enzyme at the single-cell level by recording bioluminescence images of transfected HepG2 cells. After D-LH_2_ addition, bioluminescence was recorded at the peak time using a 3-min exposure (Fig. 9, *A*–*C*). Individual cells with bright and detectable signals were measured. Both variants gave higher numbers of detectable cells (WT, *n* = 17; N116H, *n* = 77; N116H/M118L, *n* = 83), showing 1.4-fold and 1.3-fold higher signal intensity for N116H and N116H/M118L, respectively, compared to WT (Fig. 9*D* and Table S5). Compared to the standard WT enzyme and commercially optimized Luc2 by Promega Corporation, the N116H variant delivered significantly higher single-cell bioluminescence imaging sensitivity under simplified, CoA-free conditions. Furthermore, while NanoLuc relies on the use of furimazine, which can elevate cellular background autofluorescence (71), and AkaLuc requires specialized synthetic luciferin analogs to achieve its near-infrared emission (72), N116H maximizes the signal intensity using conventional, low-cost D-LH_2_. This robust performance and high signal-to-background ratio at the single-cell level makes N116H highly promising for downstream biomedical research applications, including long-term cell tracking and for high-sensitivity tumor monitoring (6).

**Figure 9:**
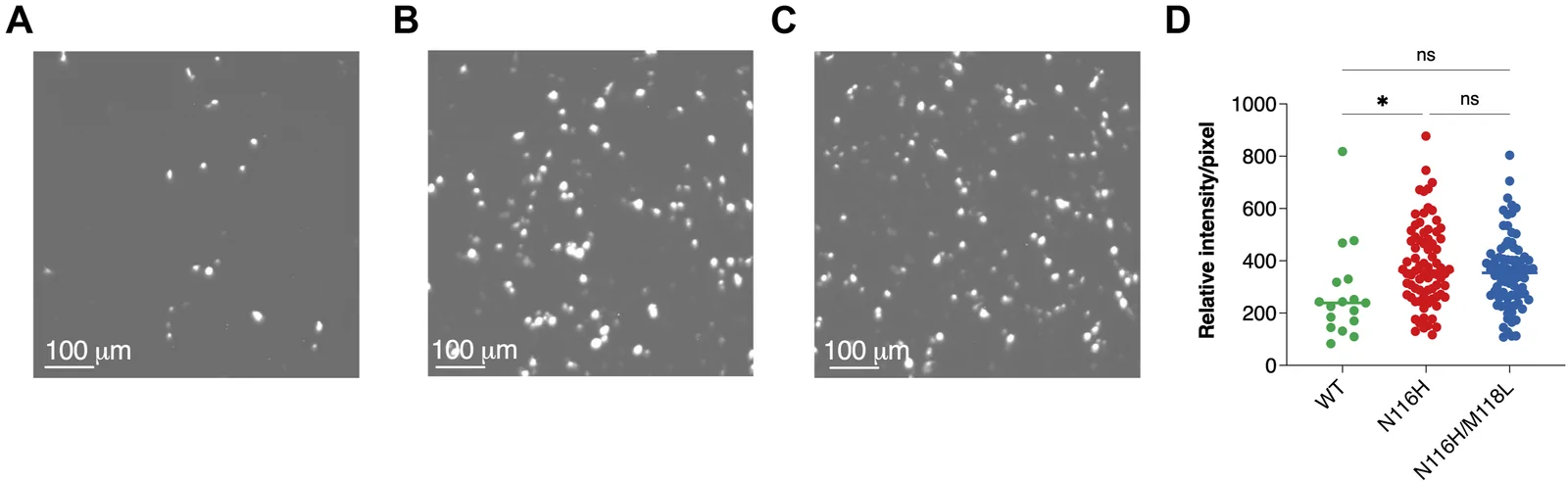
**Single cell bioluminescence imaging of Fluc variants in HepG2 cells.***A–C*, bioluminescence images of cells expressing (*A*) WT, (*B*) N116H, and (*C*) N116H/M118L acquired at the peak signal using a 3-min exposure and a 4 × objective lens (no binning; scale bar = 100 μm). Image contrast was adjusted equally. *D*, signal intensities from individual HepG2 cells expressing WT (*n* = 17), N116H (*n* = 77), and N116H/M118L (*n* = 83). Statistical significance was determined by one-way ANOVA: *p∗* < 0.01.

### Measurement of quantum yields (QY) for the N116H variant

To determine whether the enhanced bioluminescence arises from increased photon generation efficiency in addition to enhanced catalytic activity, we measured the QY following the previously reported method (16, 73, 74, 75) using the purified enzymes. Given its consistently higher bioluminescence light output across assays, N116H was selected for this analysis as a representative engineered variant. N116H exhibited a QY of 0.55 compared to the value of 0.44 for WT, corresponding to a 1.25-fold increase in QY (Table 2). This increase in QY indicates more efficient conversion of reaction energy into emitted photons, supporting the observed enhancement in bioluminescence intensity and highlighting the potential of using the N116H variant for high-sensitivity cell-based and single-cell bioluminescence imaging applications. This elevated QY reflects a more efficient conversion of chemical energy into photons, directly explaining the enhanced cellular brightness and single-cell sensitivity. At low enzyme levels, especially in high background environments, a superior QY maximizes the signal per catalytic event to lower the detection threshold. The improvement in QY of N116H may arise from the improvement in protein stability which has been reported to correlate with the improvement in luciferase performance (32). Increased structural rigidity may in turn minimize non-radiative energy loss (76) and reduce dark-pathway bifurcation, channeling a greater fraction of chemical energy into photons (75).

**Table 2:** Summary of quantum yield (QY) values and related parameters of the reactions of luciferases and D-LH_2_

Enzyme	[D-LH_2_] (nM)	Sample volume (L)	Number of D-LH_2_ molecules (x 10^11^)	Total counts (x 10^7^)	Coefficient (photon /count)	Photon^a^ (x 10^11^)	Quantum yield (QY)	λ_max_, nm
WT	1.0	0.001	6.02	9.24	2765	2.63	0.44 ± 0.04	564
N116H	0.5	0.001	0.60	1.24	2834	0.35	0.55 ± 0.04	564

a Is average of accumulated total number of photons emitted from the concentration range (6.02–60.2 × 10^10^) of reacted D-LH_2_.

## Discussion

Using semi-rational engineering combining computationally guided and site-saturation mutagenesis, we have identified N116H and N116H/M118L as functionally improved Fluc variants with higher *k*_cat_ values (light output) (Table 1 and Fig. S5), improved soluble expression (Table 1 and Figs. S5 and S6), longer half-lives at 37 °C and higher *T*_m_’s (Table 1 and Fig. S8) than the WT enzyme. Another potential advantage is their comparatively lower dependence on CoA, as both variants can produce strong bioluminescence with reduced CoA requirement (Fig. 4*D*), unlike the WT enzyme which requires CoA to enhance the signals by relieving product inhibition (Fig. 4*E*). These properties allow development of reduced-cost assay reagents for N116H or N116H/M118L in bioreporter applications (Tables S4 and S5). Their practical utility was explored in cellular systems (Fig. 6) and in real-time monitoring (Fig. S16), demonstrating more sensitive detection at the single-cell level (Fig. 9) than the WT enzyme (77). The developed bioreporter systems were also validated for use with the Keap1-Nrf2-ARE pathway in which the N116H variant showed relatively greater responsiveness to pathway activation (Fig. 8). Together, these results suggest that the engineered variants may provide improved performance across biochemical, biophysical, and cellular applications, potentially supporting their use for sensitive and cost-effective bioluminescence assays.

Unlike most of the previously reported variants in which improved thermostability came with the trade-off of reduced catalytic performance (31, 32, 33), the N116H and N116H/M118L variants preserved, and even showed improvements in the important functions of Fluc. Mutations that enhance thermal stability (*T*_m_) or prolong half-life frequently can rigidify the active site to an extent that compromises catalytic turnover (*k*_cat_). This stability and activity trade-off is illustrated by the previously reported thermostable Fluc variants Mutant E (T214A, A215L, I232A, F295L, and E345K) and YY5, a derivative of Mutant E containing additional mutations (I423L, D436G, and L530R) (31). Although these variants exhibit improved structural robustness, they suffer from substantially reduced bioluminescence output (Fig. S17). In contrast, the overall bioluminescence light outputs of N116H and N116H/M118L were much higher than those of Mutant E and YY5 (Fig. S17*B*). This may explain why although Mutant E and YY5 are thermally stable, their use in bioreporter applications still remained limited (77).

Because the mutations in N116H and M118L lie on the protein surface, far from the active site, it is unlikely that they disturb the light-emitting microenvironment. We found that the WT and N116H variants displayed nearly identical pH-dependent bioluminescence spectra, including the same red shift under acidic conditions (Fig. S18). These findings support the interpretation that the surface mutation does not interfere with the active-site environment of Fluc. This distal stabilization increases thermal stability and half-life while preserving the active site flexibility needed for efficient catalysis. By overcoming the classic stability-activity trade-off through remote rigidification, these engineered variants outperform traditional state-of-the-art enzymes, making them ideal for both robust industrial applications and high-sensitivity biological imaging.

Our investigation into the effects of CoA on N116H and N116H/M118L reactions revealed the reduced necessity for CoA in enhancing their reactions than the WT enzyme. Unlike the WT enzyme, which requires CoA for enhancing its signals (peaking at 2.4 mM) (Fig. 4*A*), the engineered variants exhibited superior bioluminescence intensity in the absence of CoA. Specifically, the LOD for N116H (0.05 nM) and N116H/M118L (0.09 nM) were lower than that of WT (0.13 nM) under the no-CoA conditions (Fig. S10). This directly indicates that these variants maintain a higher proportion of active enzyme after the first turnover (Fig. 5), translating to an optimized steady-state quantum yield where chemical energy is efficiently channeled into photon emission rather than being lost to non-radiative decay. Pre-steady-state kinetics investigation using stopped-flow techniques also confirmed this conclusion. The WT reaction showed rapid light generation (0.002–0.229 s) followed by a decay phase (0.229–0.993 s) during a first turnover and a steady-state phase after this period (Fig. 4*D*). The addition of 0.1 mM CoA increased the light output during the steady-state phase (Fig. 4*E*), confirming the thiolytic rescue of the inhibited enzyme fraction as described by Fraga *et al.* (22, 23). In contrast, the reactions of N116H and N116H/M118L variants maintained higher light levels during both the first turnover and the subsequent steady-state period even without CoA (Fig. 4*D*). These data imply that the catalytic cycle of N116H and N116H/M118L bifurcate less into the dark path when compared to WT (Fig. 5). The reduced formation of products from the non-productive pathway in these variants thus decreases their reliance on CoA for enzyme reactivation (Fig. 5), which is beneficial for reducing the use of expensive CoA in Fluc assays.

Currently, the mechanism by which the N116H and N116H/M118L variants exhibit less dependency on light enhancement by CoA is not clearly understood. Molecular docking of CoA to WT and both variants of Fluc revealed highly similar binding modes of CoA to all enzymes (Fig. S19), indicating that the mutations do not disrupt the overall CoA-binding site. This suggests that the reduced light enhancement by CoA in the variant reactions unlikely arises from impaired CoA recognition. The transient kinetic data showed that in the absence of CoA, the variants exhibited a decrease in partitioning into the non-productive pathway, possibly due to lowered accumulation of the inhibitory dehydroluciferyl-adenylate intermediate, which thus necessitates CoA-mediated reactivation (Figs. 4 and 5). Altogether, the current data suggest that the enhanced CoA-independent activity of the variants arises primarily from altered catalytic pathway partitioning, rather than from changes in the CoA binding site.

Our results demonstrate that the engineered Fluc variants can be effectively applied in a dual-reporter system by combining them with FLUX^Vc^ (Figs. 6 and S12 and 13), providing a cost-effective alternative to the conventional Fluc/Rluc system (Tables S3 and S4). The Fluc/FLUX^Vc^ system showed comparable performance in signal normalization while significantly reducing assay cost due to the use of bacterial luciferase and simpler substrates. This is consistent with previous studies reporting the use of FLUX^Vc^ as an alternative internal control in mammalian cells (36), where it provides stable and reliable signals with lower cost compared to Rluc. The advantage of the Fluc/FLUX^Vc^ system is further enhanced by the reduced CoA dependence of the engineered Fluc variants, allowing the use of simplified assay reagents without compromising signal intensity. Single-cell bioluminescence imaging is often limited by low enzyme levels, high background noise, and rapid signal decay. Because our engineered Fluc variants maintain strong bioluminescence without needing extra CoA, they also simplify the intracellular bioluminescence measurement. Optimal signal-to-background ratio and tracking time can be achieved without needing CoA supplementation which could otherwise result in cell toxicity. We validated this system using the Keap1-Nrf2-ARE pathway, a well-established model for oxidative stress response widely used in bioreporter assays (46, 47, 48, 66, 67, 68). Upon treatment with carnosic acid, a known Nrf2 activator (46, 47, 67, 68), N116H showed stronger and more sensitive responses compared to WT (Fig. 8), indicating improved detection capability. Because similar reporter-based approaches have been widely used to study transcriptional regulation and drug screening (66, 67, 78), the enhanced light output of N116H and N116H/M118L variants would support them to be used as a robust, sensitive, and economical platform for cellular bioreporter applications and HTS.

In conclusion, the Fluc variants with improved functions identified in this work, particularly N116H, are promising enzymes that can serve as versatile and efficient bioluminescence platforms with strong potential for high-throughput screening, cellular imaging, and quantitative bioreporter applications. Despite these advantages, a few limitations remain. The Fluc variants emit green-yellow light (∼560 nm), which works well for *in vitro* assays but suffers from tissue absorption and scattering during deep-tissue *in vivo* imaging. Future studies will focus on characterizing the underlying biophysical mechanisms that allow these surface mutations to prevent product inhibition. Additionally, we plan to integrate these enzymes into automated HTS pipelines to fully exploit their cost-effectiveness on an industrial scale.

## Experimental procedures

### Chemicals and reagents

All commercially available chemicals were purchased from Agilent, Sigma-Aldrich, TCI, Merck, or Thermo Fisher Scientific. Molecular biology reagents including T4 DNA ligase, restriction enzymes, PCR kits, and Gibson assembly kits were obtained from New England Biolabs (NEB), Takara Bio, and Thermo Fisher Scientific. Plasmid extraction kits and Gel/PCR purification kits were purchased from Favorgen and Takara Bio. Human cell lines—HEK293T (human embryonic kidney) cell, HepG2 (human hepatocellular carcinoma) cell, and HeLa (human cervical adenocarcinoma) cell—were obtained from the American Type Culture Collection (ATCC) and RIKEN Cell Bank (RCB). Cell culture media, trypsin, penicillin-streptomycin, and fetal bovine serum (FBS) were purchased from Thermo Fisher Scientific. The Fluc assay system (E1500) and Rluc assay system (E2810) and reagents were obtained from Promega Corporation. Oligonucleotide primers were synthesized by Integrated DNA Technologies (IDT). DNA sequencing services were provided by ATCG Co., Ltd, U2Bio, and Eurofins.

### FireProt analysis: Semi-rational design of Fluc variants

Thermostable variants were predicted using FireProt 1.0 (49, 79, 80). The crystal structure of Fluc (PDB ID: 4G36) was retrieved from the Protein Data Bank (PDB) and used as an input for prediction. FireProt 1.0 was run using default parameters. Candidate residues were selected based on surface location without applying ΔΔG stability cutoffs. Structural analysis was conducted using both sequence conservation data and structure-based energy minimization by Rosetta and FoldX to identify optimal multiple-point mutants (49, 79, 80). PyMOL 2.5 (Schrödinger) (https://pymol.org) was used for structural visualization.

### Construction of Fluc variants

The Fluc WT was constructed accordingly. The *P. pyralis* luciferase gene (*luc+)* (GenBank accession number: U47296.2) was codon-optimized for expression in *E. coli* and synthesized by GenScript Biotech Corporation. The codon-optimized luciferase gene was then cloned into the pET11a vector at *Hind*III and *Xba*I sites, and the resulting recombinant plasmid (pET11a-*Fluc*) (36, 44, 45) was transformed into competent *E. coli* XL1 blue cells (Thermo Fisher Scientific) for Fluc expression (36, 44, 45).

Site-directed mutagenesis was carried out using the pET11a-*Fluc* plasmid as a template according to QuikChange site-directed mutagenesis protocol (Agilent Technologies). Mutations were confirmed by DNA sequencing (IDT Singapore).

To generate Fluc site-saturation mutagenesis libraries of the selected candidates, degenerate primers containing NNK codons at the target residues were designed (Table S2) and used in PCR reactions (37, 81). The library was transformed into *E. coli* BL21 (DE3) for plasmid propagation and subsequently extracted (Favorgen) for DNA sequencing. *E. coli* XL-1 Blue was initially tested; however, comparable library quality was obtained in both strains. Therefore, *E. coli* BL21 (DE3) was used to maintain a consistent host for both plasmid propagation and downstream expression screening. The nucleotide distribution of each library was evaluated using the *Q*_*pool*_ value, and libraries with a *Q*_pool_ of ≥ 0.7 were selected for further screening (82).

### Library screening of enzyme variants using a high-throughput screening platform

One hundred nanograms of each plasmid library were transformed into *E. coli* BL21(DE3), and the transformants were selected using LB agar containing 50 μg/ml ampicillin. Colony picking and bacterial culturing were done using a colony picker (K6 Biosystems) and liquid handling systems (PerkinElmer Explore G3 Integrated Workstation). Each colony was picked and grown in a 200 μl ZYM-5052 medium containing 50 μg/ml ampicillin in 96-well plates at 37 °C for 16 h. Subsequently, a 6 μl of starter culture was inoculated into 194 μl of fresh ZYM-5052 medium supplemented with 50 μg/ml ampicillin and grown at 37 °C for 6 h (OD_600_ = 0.2–0.4). Fluc expression was induced at 25 °C for 16 h. Cells were harvested by centrifugation at 2700*g* for 1 h and then lysed with 100 μl lysis buffer (20 mM sodium phosphate pH 7.0, 5 mM DTT, 1 mM phenylmethylsulfonyl fluoride (PMSF), 5 mM EDTA, 2 mg/ml lysozyme, and 0.1% Triton X-100) by shaking at 25 °C for 2 h. The resulting supernatant was incubated at 45 °C for 30 min to impose thermal selection pressure for identifying thermostable variants, and the denatured protein was removed by centrifugation at 2700*g* for 1 h. Then, the supernatant (50 μl) was added to a 96-well black plate (PerkinElmer) and the reaction was initiated by injecting 50 μl of a substrate solution (100 μM D-LH_2_, 6 mM ATP, and 6 mM MgCl_2_ in 100 mM HEPES, pH 8.0), and the reaction was carried out at 25 °C. Bioluminescence signals were recorded for 30 s using an EnVision microplate reader (PerkinElmer). Clones showing activity greater than that of the WT enzyme were selected for DNA sequencing.

### Expression and purification of Fluc

Fluc WT and variants were expressed in *E. coli* BL21(DE3). Cells were transformed with constructed plasmids. A single colony of transformant was grown in ZYM-5052 broth supplemented with 50 μg/ml ampicillin at 37 °C to generate a starter culture. The starter culture was inoculated (equivalent to 1% of a final culture volume) in a fresh ZYM–5052 broth supplemented with 50 μg/ml ampicillin and grown until the OD_600_ reached 0.8 to 1.0. The culture was then incubated at 25 °C for 16 h to induce protein expression, and cells were harvested by centrifugation at 11,000*g* for 10 min. Cell pellets were resuspended in buffer A (50 mM sodium phosphate (pH 7.0) containing 1 mM DTT, 60 μM PMSF, and 0.3 mM EDTA) and lysed by sonication. Cell debris was removed by centrifugation at 25,000*g* for 30 min.

For protein purification, a two-step precipitation using 0.1% PEI and ammonium sulfate fractionation was initially carried out. Crude extract was added with PEI to a final concentration of 0.1% (w/v) at 4 °C for 30 min, and the suspension was centrifuged at 25,000*g* to remove nucleic acids. The resulting supernatant was subjected to ammonium sulfate fractionation at 0 to 30% and 30 to 60% saturation at 4 °C. After centrifugation at 25,000*g*, the 30 to 60% (w/v) ammonium sulfate precipitated protein fraction was resuspended in 25 mM sodium phosphate (pH 7.0) and dialyzed overnight against 4 L of the same buffer using a 10 kDa molecular weight cutoff dialysis bag (Thermo Fisher Scientific). The dialyzed protein was centrifuged at 25,000*g* for 30 min, and the supernatant was applied onto DEAE-Sepharose Fast Flow chromatography (1.5 cm × 27 cm). The protein was eluted using a linear gradient of sodium chloride (NaCl, 0–300 mM) in 25 mM sodium phosphate (pH 7.0). Fractions containing Fluc (62 kDa) identified by SDS-PAGE (Fig. S6) were pooled and concentrated using Amicon stirred cells and a 10 kDa molecular weight cutoff membrane. The purified Fluc was exchanged into 25 mM Tris-HCl (pH 7.0) containing 20% glycerol using Sephadex G-25 gel filtration column and stored at −80 °C until use.

For certain experiments where a partially purified Fluc was used, the Fluc variant was prepared using a two-step precipitation of 0.1% PEI and ammonium sulfate fractionation as described above.

### Thermostability assays

Thermostability was evaluated using both residual activity measurements and fluorescence-based thermal shift assays. For activity-based assessments, enzyme solutions were subjected to thermal stress, immediately cooled on ice, and centrifuged at 2700*g* for 10 min to remove denatured proteins. After that, bioluminescence was recorded for 30 s using an AB-2270 single-tube luminometer (ATTO). Specific incubation and assay conditions varied depending on the experiment. For screening of hit variants, partially purified enzyme (10 μM) was incubated at 45 °C for 20 min and assayed with a reaction mixture containing 100 μM D-LH_2_, 3 mM ATP, and 6 mM MgCl_2_. For studying activities of candidate variants, purified variant (2.14 nM) was incubated at 45 °C for 10 min and the activity was measured using 25 μM D-LH_2_, 2 mM ATP, and 2 mM MgCl_2_ in 100 mM HEPES (pH 8.0).

To determine the half-life (*t*_1/2_) of variants, purified variants (10 μM) were incubated at 37 °C for 6 h. During the incubation, the activities of the enzymes were measured at different time points using 25 μM D-LH_2_ in 100 mM Tris-HCl (pH 8.0). The data were fitted to a one-phase exponential decay model using *GraphPad Prism* version 10.6.1, GraphPad Software, www.graphpad.com.

Melting temperatures (*T*_m_) of variants were determined using ThermoFluor assays by mixing purified enzymes with SYPRO Orange dye (83, 84) and ramping the temperature from 25 °C to 95 °C at 1 °C increments in a CFX Connect real-time PCR thermocycler (Bio-Rad). Final reaction mixtures contained 40 μM enzyme with 20X dye in 100 mM sodium phosphate buffer (pH 7.0). The *T*_m_ values were calculated from the inflection point of the resulting fluorescence curves using *GraphPad Prism* version 10.6.1.

### Steady-state kinetics of Fluc variants with D-LH_2_

Reactions (150 μl, 100 mM Tris-HCl pH 8.0) were performed at 25 °C using an AB-2270 single-tube luminometer (ATTO). Bioluminescence was initiated by adding 125 μl of 3 mM ATP and 6 mM MgCl_2_ to 25 μl of premixed luciferase with D-LH_2_ (0–50 μM). Signals were recorded for 180 s and integrated to obtain the total light output, which was divided by 180 s to give the average bioluminescence intensity (BLI), expressed as counts per second (CPS). Because BLI is proportional to the product of quantum yield, *k*_cat_, and enzyme concentration (BLI = quantum yield × *k*_cat_ × [E]) (73), CPS values were divided by the fixed amount of Fluc used in each reaction to obtain a relative *k*_cat_ term (quantum yield × *k*_cat_), expressed in units of CPS·nM^-1^. These enzyme-normalized BLI values at varying D-LH_2_ concentrations were fit to the Michaelis–Menten equation using nonlinear regression in *GraphPad Prism* version 10.6.1 to obtain the relative *k*_cat_ and *K*_m_ values.

### Effects of various additive compounds on Fluc activity

Effects of different additives−EDTA, DTT, BSA, and CoA−on the luciferase activities of the WT and variants were assessed. The concentration ranges for each additive were EDTA (0–10 mM), DTT (0–20 mM), BSA (0–10 mg/ml), and CoA (0–3.2 mM). The purified variant (1 μM) was incubated with each additive at varying concentrations for 10 min. Then, the bioluminescence reaction was initiated by adding 150 μl of assay mixture containing 25 μM D-LH_2_, 3 mM ATP, and 6 mM MgCl_2_ in 100 mM Tris-HCl (pH 8.0) to the 5 μl enzyme-additive mixture. Bioluminescence intensity was measured over 30 s using an AB-2270 single-tube luminometer (ATTO).

### Investigation of coenzyme A effects on bioluminescence kinetics and signal linearity

Bioluminescence kinetics of WT and its variants were performed using an SF spectrometer (model SF-61DX, TgK Scientific) in single-mixing mode, placed inside an anaerobic glove box. All reactions were carried out in triplicate under a light-off mode at 25 °C with 100 mM Tris-HCl (pH 8.0). The reactions were initiated by mixing equal volumes of two oxygenated solutions, one containing the reaction mixture (80 μM D-LH_2_, 6 mM ATP, 12 mM MgCl_2_, and 0.2 mM CoA) and the other containing 0.50 μM Fluc enzyme. The final concentrations after mixing were half of that described. Bioluminescence traces were monitored and recorded by measuring fluorescence emission with an excitation wavelength of λ_ex_ ≥ 325 nm. The photomultiplier tube was maintained at a constant voltage of 700 mV, and data were collected for 2000 s.

To investigate bioluminescence linearity, activity assays were performed using an AB-2270 single-tube luminometer (ATTO). A two-fold serial dilution of luciferase (starting from 1 μM) was prepared. Reactions were initiated by adding 100 μl of assay cocktail (25 μM D-LH_2_, 3 mM ATP, and 6 mM MgCl_2_ in 100 mM Tris-HCl, pH 8.0) to the diluted enzyme solution. This study was performed under two conditions: in the absence of CoA and in the presence of 0.1 mM CoA. Bioluminescence was recorded for 20 s following a 5 s delay.

### Bioluminescence emission spectra of Fluc variants

Bioluminescence emission spectra of WT and its variants (N116H and N116H/M118L) were monitored by mixing 10 μl of 2 μM purified enzyme with 10 μl of substrate mixture (3 mM ATP, 6 mM MgCl_2_ and 25 μM D-LH_2_ in100 mM Tris-HCl, pH 8.0). Emission spectra were recorded using a highly sensitive CCD spectrophotometer (ATTO AB-1850s, Lumi Fluoro Capture) with a 5 s shutter time and 1 mm slit width.

For investigating the effects of pH on the bioluminescence spectra of WT and N116H, the following experiments were carried out. Reactions (150 μl total volume) were prepared in a 0.2 ml PCR tube by combining 115 μl of a tri-buffer system (85) (40 mM MES, 40 mM Tris, and 40 mM sodium phosphate, pH-adjusted with HCl/NaOH to the indicated pH: 6.0, 6.5, 7.0, 7.5, or 8.0), 5 μl of 90 mM ATP, 5 μl of 180 mM MgCl_2_, and 20 μl of 150 μM D-LH_2_. The reaction was initiated by adding 5 μl of 1.8 μM purified enzyme, yielding final concentrations of 3 mM ATP, 6 mM MgCl_2_, 20 μM D-LH_2_, and 60 nM luciferase. Immediately after enzyme addition, the open PCR tube was placed directly into the light-tight sample chamber of the CCD spectrophotometer (ATTO AB-1850s, Lumi Fluoro Capture), which is fitted with a holder designed to accept 0.2 ml PCR tubes and position them within the optical path of the detector. Emission spectra were recorded at room temperature using a shutter time of 2 s and a slit width of 1 mm. All spectra were normalized to their respective maximum emission intensity (set to 1.0) to enable direct comparison of spectral profiles across pH conditions.

### Construction of plasmids for expression in mammalian

The mammalian cell expression vectors pGL4-SV40*-N116H* and pGL4-SV40*-N116H/M118L* were constructed based on the pGL4 plasmid which contains the *luc2* (WT) gene regulated by the SV40 promoter (Promega Corporation) as illustrated in Figure S11*A*. Site-directed mutagenesis was performed to introduce the *N116H* and *N116H/M118L* mutations using the QuikChange site-directed mutagenesis protocol (Agilent Technologies) and the primer pairs listed in Table S2.

For the ARE-responsive reporters, the plasmid pGL4-ARE-minP-*Fluc* was constructed *via* Gibson Assembly as illustrated in Figure S11*C*. A linearized backbone was generated by site-directed mutagenesis of the pGL4.10-*N116H* and pGL4.10-*N116H/M118L* vector. DNA fragments of the ARE fused to a minimal promoter (minP) were synthesized by Integrated DNA Technologies (IDT, Singapore). These fragments were PCR-amplified with overlapping ends and assembled with the linearized vector.

To serve as internal control reporters, two additional plasmids were prepared. The pGL3-TK- *FLUX*^*Vc*^ vector was constructed by removing the NF-κB response element from the pGL3-NF-κB-TK*- FLUX*^*Vc*^ plasmid (36). The parent vector was digested with *Spe*I and *Sac*I, created blunt end by adding T4 DNA polymerase (NEB) in the presence of dNTPs and re-ligated using T4 DNA ligase (NEB). However, due to the lack of detectable activity with the TK promoter in alternative experimental settings, a second internal control utilizing the CMV promoter was then developed. This plasmid, pcDNA3-*FLUX*^*Vc*^ plasmid was assembled using the In-Fusion HD Cloning Kit (Takara Bio). The pcDNA3 vector was linearized *via* PCR, and the *FLUX*^*Vc*^ gene was amplified from pGL3-NF-κB-TK*- FLUX*^*Vc*^ (36) using primers as listed in Table S2. The vector and insert were assembled at a 1:2 M ratio.

All constructs were validated by DNA sequencing services provided by U2Bio (Thailand) or Eurofins Genomics (Japan).

### Cell culture

HEK293T cells (CRL-3216, ATCC) and HeLa cells (RCB0007, RIKEN) were cultured in Dulbecco’s Modified Eagle Medium (DMEM, FUJIFILM Wako Pure Chemical Corporation) supplemented with 10% heat-inactivated fetal bovine serum (FBS, Merck).

HepG2 cells (RCB1886, RIKEN) were cultured under the same condition as described for HEK293T and HeLa, but with the addition of 1x non-essential amino acids (NEAA, Gibco) and 1 mM sodium pyruvate (Gibco).

All cell lines were maintained at 37 °C in a humidified incubator under an atmosphere of 5% CO_2_.

### Evaluation of cell lysis conditions for bioluminescence assays

The optimal cell lysis volume of the in-house lysis reagent (50 mM sodium phosphate (pH 7.0), 1% (w/v) CHAPS, 1 mM EDTA, and 10% (v/v) glycerol) was determined using HEK293T cells, which were selected as the initial assay—development platform because of their high transfection efficiency and robust reporter expression. HEK293T cells (1 × 10^5^ cells/well) were seeded in a 24-well plate 1 day prior to transfection for testing. Cells were co-transfected with 200 ng of pGL4-SV40-*Fluc* (WT) and 20 ng of pGL3-TK-*FLUX*^*Vc*^ using Lipofectamine 3000 (Thermo Fisher Scientific). After a 6 h incubation at 37 °C, the medium was replaced with Dulbecco’s Modified Eagle Medium (DMEM) supplemented with 10% (v/v) FBS. The cells were cultured for an additional 24 h at 37 °C in a humidified atmosphere containing 5% CO_2_ to allow for luciferase expression. Cells were then washed with PBS and lysed using either 100 μl or 200 μl of the in-house lysis buffer.

Fluc activity was determined by injecting a cocktail containing 25 μM D-LH_2_, 3 mM ATP, and 6 mM MgCl_2_ in 100 mM Tris-HCl (pH 8.0) into 4 μl of cell lysate. FLUX^Vc^ activity was measured by adding 100 μl of a reaction mixture containing 5 μM FMN, 100 μM HPA, 10 μM decanal, and 100 μM NADH to 4 μl of lysate pre-mixed with 50 mU of C_1_ reductase. Bioluminescence signals were recorded using an AB-2270 single-tube luminometer (ATTO) with a 5 s delay followed by a 20 s measurement. Fluc signals were normalized to FLUX^Vc^ activity.

Additionally, the lysis efficiencies of in-house and passive lysis buffer (PLB) were compared. The buffer volume used was 100 and 200 μl. The experiments were performed as described above with the two buffers running in parallel.

### Characterizations of bioluminescence intensity and emission spectra in mammalian cells

HEK293T, HeLa, and HepG2 cells carrying dual reporter system of Fluc/FLUX^Vc^ and Fluc/Rluc (prepared as described in the section “*Evaluation of cell lysis conditions for bioluminescence assays”*) were lysed using 200 μl in-house lysis buffer and the activities were quantified using a luminometer as described previously.

For bioluminescence emission spectra analysis, cells were seeded in 35 mm dishes (1 × 10^6^ cells/dish), transfected with 200 ng of reporter plasmid, and subjected to a medium replacement 6 h post-transfection. Following a 24 h incubation, cells were lysed in an increased volume of 500 μl, and spectral data were recorded using a high-sensitivity CCD spectrophotometer (AB-1850s Lumi Fluoro Capture, ATTO) with a 180 s shutter time and 1 mm slit width by mixing 20 μl of lysate with 20 μl of assay buffer (25 μM D-LH_2_, 3 mM ATP, and 6 mM MgCl_2_ in 100 mM Tris-HCl, pH 8.0).

### Monitoring of the Keap1-Nrf2-ARE signaling pathway using Fluc/FLUX^Vc^ system

To evaluate the Keap1-Nrf2-ARE signaling pathway, reverse transfection was performed in 96-well plates. Transfection complexes were first prepared by mixing 200 ng of the Fluc reporter plasmid (pGL4-ARE-minP*-Fluc* (WT, N116H, or N116H/M118L)) and 20 ng of the internal control reporter plasmid (pcDNA3*-FLUX*^*Vc*^) with Lipofectamine 3000 in Opti-MEM. After a 30 min incubation at 37 °C to allow complex formation, HepG2 cells were added to the wells at a density of 1.5 × 10^4^ cells/well. At 24 h post-transfection, the culture medium was replaced with DMEM supplemented with 10% v/v FBS, 1 × NEAA, and 1 mM sodium pyruvate, containing a 2-fold serial dilution of carnosic acid starting at 100 μM.

Cell lysis and bioluminescence measurements were subsequently performed as described in the section “*Evaluation* *of cell lysis conditions for bioluminescence assays.*” Fluc signals were normalized to the internal control (FLUX^Vc^) activity, and the resulting ratios were further normalized to the vehicle control (0.1% DMSO) to calculate fold-change. Data were fitted using nonlinear regression in *GraphPad Prism* 10.6.1.

### Real-time bioluminescence monitoring and single-cell bioluminescence imaging in HepG2 cells

To conduct real-time bioluminescence monitoring, reverse transfection was performed in 96-well white clear-bottom plates (Nunc, Thermo Fisher Scientific). Transfection complexes were prepared as described in the section “*Monitoring of the Keap1-Nrf2-ARE signaling pathway using Fluc/FLUX*^*Vc*^ *system*,” to contain 200 ng of reporter plasmids (pGL4-SV40*-Fluc-*WT, N116H, or N116H/M118L). HepG2 cells were subsequently added to the wells at a density of 5 × 10^4^ cells/well. At 24 h post-transfection, the culture medium was replaced with phenol red-free DMEM supplemented with 10% (v/v) FBS, 1 × NEAA, 1 mM sodium pyruvate, and 100 μM D-LH_2_. Bioluminescence was recorded at 37 °C every 30 min over a 72-h period using a WSL-1565 Kronos HT luminometer (ATTO), with an integration time of 10 s per measurement.

For single-cell bioluminescence imaging, HepG2 cells (1 × 10^6^ in 35 mm dishes) were forward transfected with 200 ng of reporter plasmid. At 6 h post-transfection, the medium was replaced with DMEM containing 25 mM HEPES (pH 7.0). After 24 h, the medium was replaced with phenol red-free DMEM containing 200 μM D-LH_2_. Bioluminescence was recorded every 10 min with 1 min interval time for 17 h using an AB-2550 Kronos Dio luminometer (ATTO); upon reaching peak intensity, cells were imaged with a 3 min exposure using an AB-3000B Cellgraph. Image contrast was adjusted equally across all samples and bioluminescence intensity for individual cells was quantified using *Cellgraph Viewer* software (ATTO). All data were analyzed using one-way ANOVA in *GraphPad Prism* 10.6.1.

### Quantum yield (QY) determination

QY of WT and N116H were determined as reported previously (16, 73, 74, 75). All solutions were prepared in 100 mM Tris-HCl (pH 8.0). The reaction (200 μl) contained 0.5 to 5 nM D-LH_2_, 3 mM ATP, and 3 mM MgCl_2_. Prior to adding the enzyme, the reaction mixture was placed in the luminometer to record background signals. The reaction was initiated by injection of 20 μl of 5 μM purified luciferase. Bioluminescence was monitored until the signal decayed to baseline, and the QY was calculated from the total integrated photon emission. To enable absolute photon quantification, the luminometer was calibrated against a sphere spectrometer equipped with a cooled CCD detector and a reference light source. A linear standardization curve was established to correlate the luminometer's relative count values with the absolute photon flux measured by the spectrometer.

### Molecular docking of CoA in Fluc WT and Fluc variants

The crystal structure of Fluc in the adenylate-forming conformation complexed with 5′-O-[N-(dehydroluciferyl)-sulfamoyl] adenosine (DLSA) (PDB ID: 4G36) was obtained from the RCSB Protein Data Bank (86). The DLSA molecule was retained to preserve the adenylate-forming conformation and to examine its relationship with CoA. Structural models of the N116H and N116H/M118L variants were generated using CHARMM-GUI (87) by introducing the desired amino acid substitutions (88). The three-dimensional (3D) structure of CoA (PDB Chemical Component ID: COA) was retrieved from the RCSB Protein Data Bank (89). Docking was performed using GOLD (90, 91) with 100 genetic algorithm (GA) runs. The highest-ranked configuration was selected based on the Piecewise Linear Potential (PLP) fitness score. Protein–ligand interactions, including contacts between CoA and active-site residues, were analyzed and visualized in 3D using PyMOL 2.5 (Schrödinger) (https://pymol.org).

### Statistical analysis

All experiments were performed using at least three independent biological replicates, with technical triplicates for each condition unless otherwise stated. Data are presented as the mean ± standard deviation (SD). Curve fitting and statistical analyses were conducted using *GraphPad Prism* version 10.6.1. Specifically, dose–response curves were analyzed *via* nonlinear regression, and bioluminescence decay curves for half-life determination were fitted to a single exponential decay model.

For hypothesis testing, comparisons between two groups were performed using an unpaired two-tailed Student’s *t* test, while multiple group comparisons were analyzed using a one-way ANOVA followed by Tukey’s *post hoc* test. A *p*-value < 0.01 was considered statistically significant.

## Data availability

All data are contained within the manuscript or Supplementary Information.

## Supporting information

This article contains supporting information.

## Conflict of interest

The authors declare the following financial interests/personal relationships which may be considered as potential competing interests: C. K., P. W., and P. C. are inventors on a filed patent application in Thailand (Patent Application No. 2401003513) related to the enzyme variants reported in this study. The authors declare no other competing interests.
