# Supplementary material for: Biochemical, bioluminescence and bioreporter properties of functionally improved firefly luciferases
Source: J Biol Chem. 2026 Sep 18;302(11):113568. doi: 10.1016/j.jbc.2026.113568 (PMC13715313; doi:10.1016/j.jbc.2026.113568)
Supplement: Supplementary file 1 [file mmc1.pdf]

Supporting information

Biochemical, Bioluminescence and Bioreporter Properties of Functionally Improved Firefly Luciferases[#]

Chadaporn Kantiwiriyawanitch[1], Vinutsada Pongsupasa[1], Pratchaya Watthaisong[1], Jittima Phonbuppha[1], Aisaraphon Phintha[1], Daisuke Sasaki[2], Thanyaporn Wongnate[1], Ruchanok Tinikul[3], Kazuki Niwa[4], Yoshihiro Nakajima[2], Yoshihiro Ohmiya[1], Ubolsree Leartsakulpanich[5*], and Pimchai Chaiyen[1*]

Materials including **Supplementary Figures. 1-19** and **Tables. 1-5**

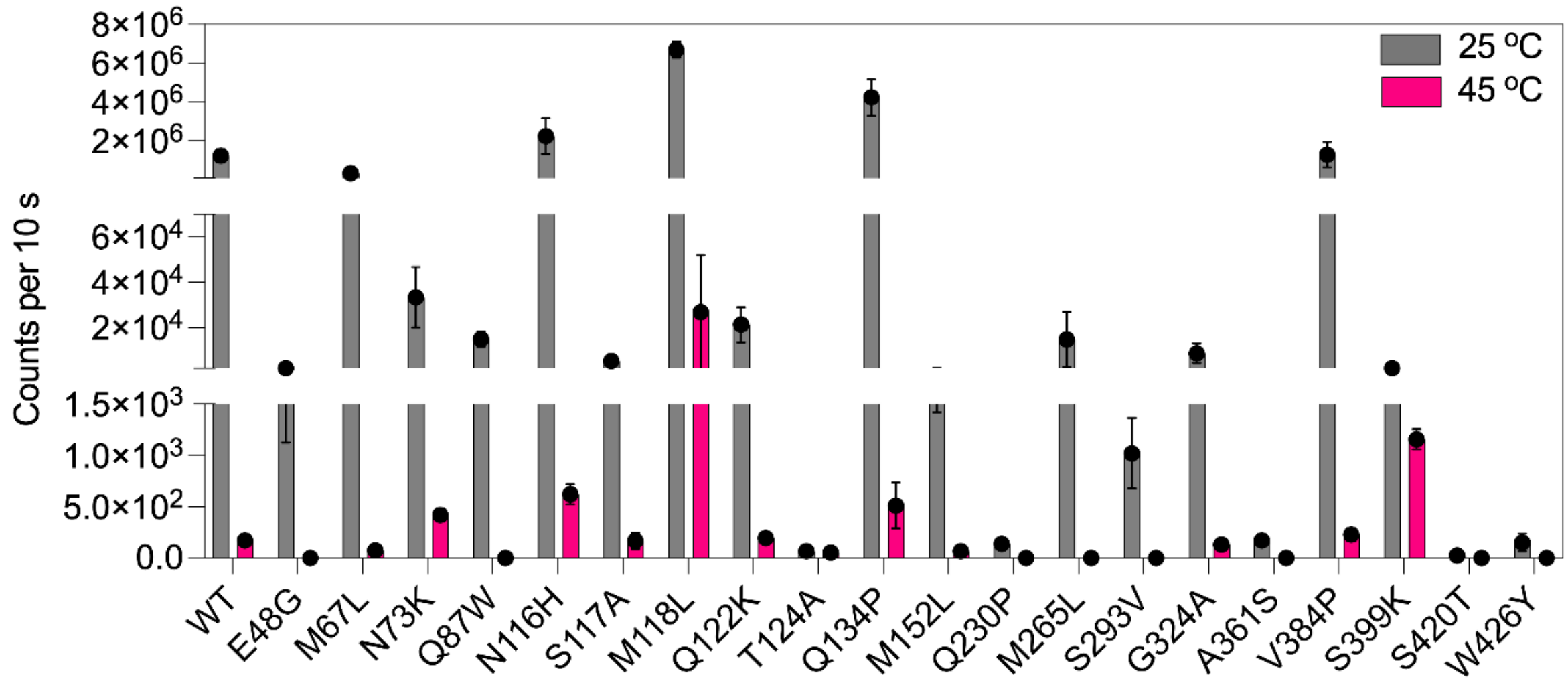

Fig. S1. Screening of selected surface variants of Fluc using crude lysates. Bioluminescence activities were measured before and after heat treatment at 45 °C for 10 min. Grey bars indicate no heat treatment whereas pink bars represent samples heated at 45 °C for 10 min. Data are presented as mean ± S.D. (n = 3).

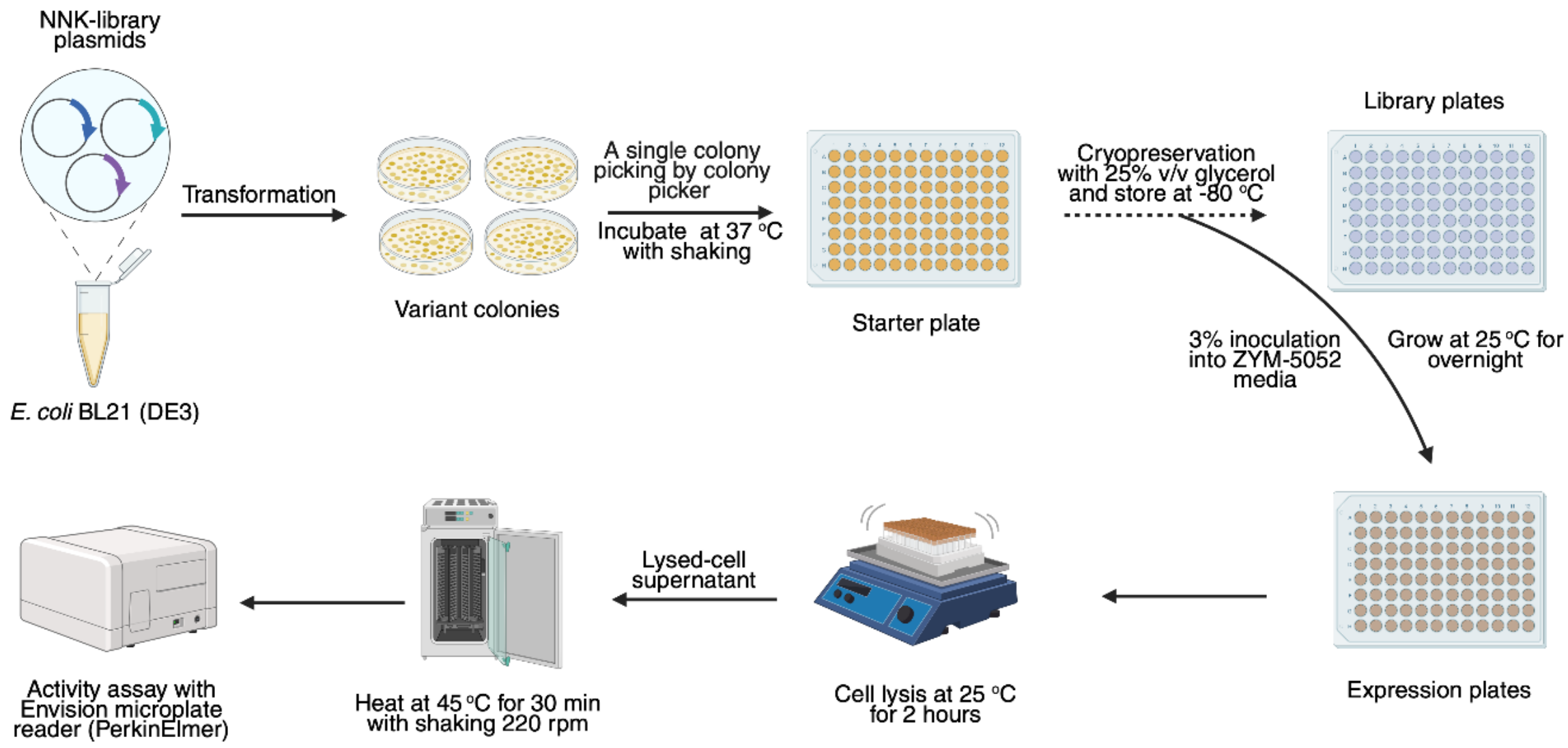

Fig. S2. A high throughput screening workflow to obtain Fluc variants with improved thermostability. Each plasmid library was transformed into *E. coli* BL21(DE3). Each single colony was picked by automation and cultured in 200 µL liquid ZYM-5052 medium containing 50 µg/mL ampicillin in 96-well plates using a colony picker (K6 Biosystems) and liquid handling systems (PerkinElmer Explore G3 Integrated Workstation). Subsequently, replica plates of the libraries were created and cryo-preserved. Each plate of replicas was used as a starter culture (6 µL each) to inoculate into 194 µL of fresh ZYM-5052 medium containing 50 µg/mL ampicillin. Cells were grown at 37 °C for 6 h (OD_{600} = 0.2–0.4) and then at 25 °C for 16 h. Cells were harvested and lysed with 100 µL lysis buffer (20 mM sodium phosphate (pH 7.0), 5 mM DTT, 1 mM PMSF, 5 mM EDTA, 2 mg/mL lysozyme, and 0.1% Triton X-100) by shaking at 25 °C for 2 h. The resulting supernatant was incubated at 45 °C for 30 min with shaking at 220 rpm. The activity of the preheated enzyme (50 µL) was then determined by mixing with 50 µL of a substrate solution (100 µM D-LH_2, 6 mM ATP, and 6 mM $MgCl_2$ in 100 mM HEPES, pH 8.0) at 25 °C and measuring bioluminescence

signals for 30 s using an EnVision microplate reader (PerkinElmer). Clones showing the highest post-heat treatment activities were selected for DNA sequencing.

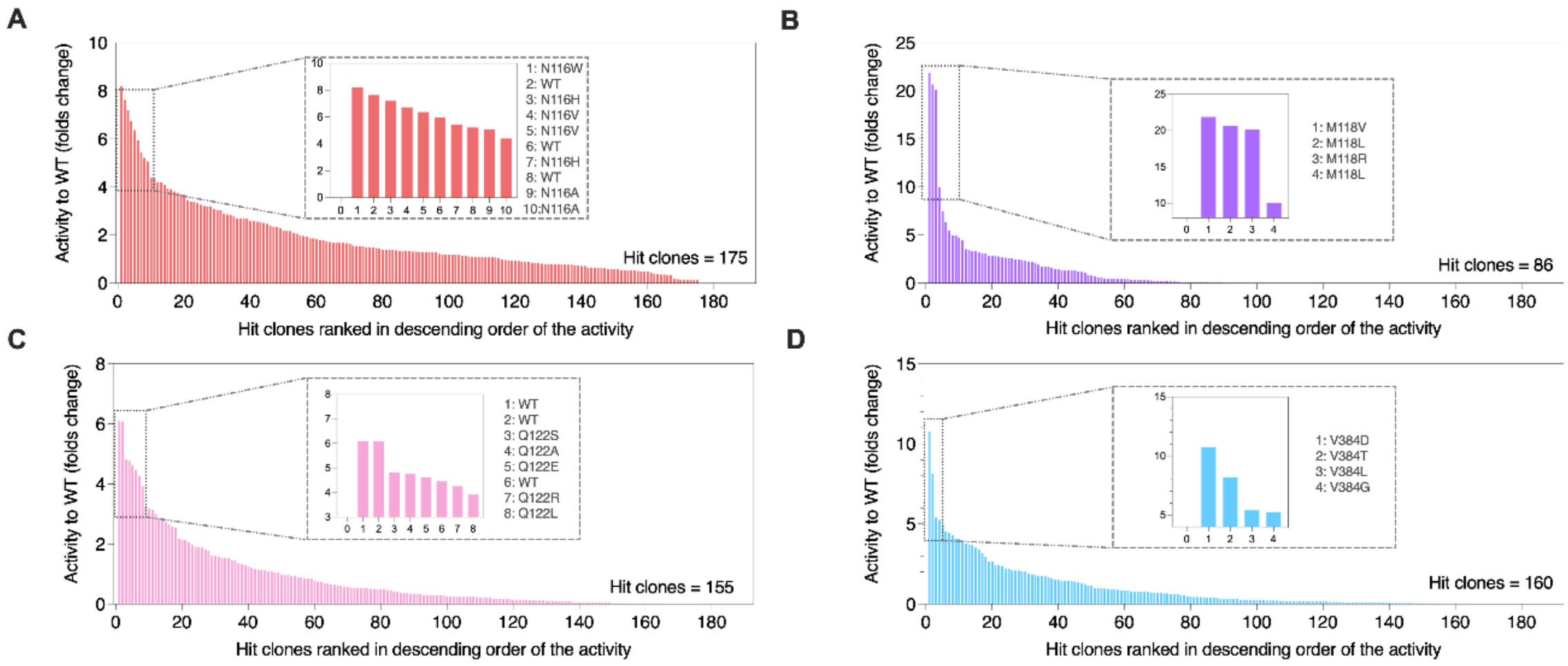

Fig. S3. Screening for thermostable Fluc variants. Four site-saturation libraries–(A) N116X, (**B**) M118X, (**C**) Q122X, and (**D**) V384X–were screened for candidate clones based on bioluminescence activities after preheating at 45 °C for 30 min. Clones showing 4- to 20-folds of activities greater than that of WT were selected for DNA sequencing. Their identities are indicated in the insets.

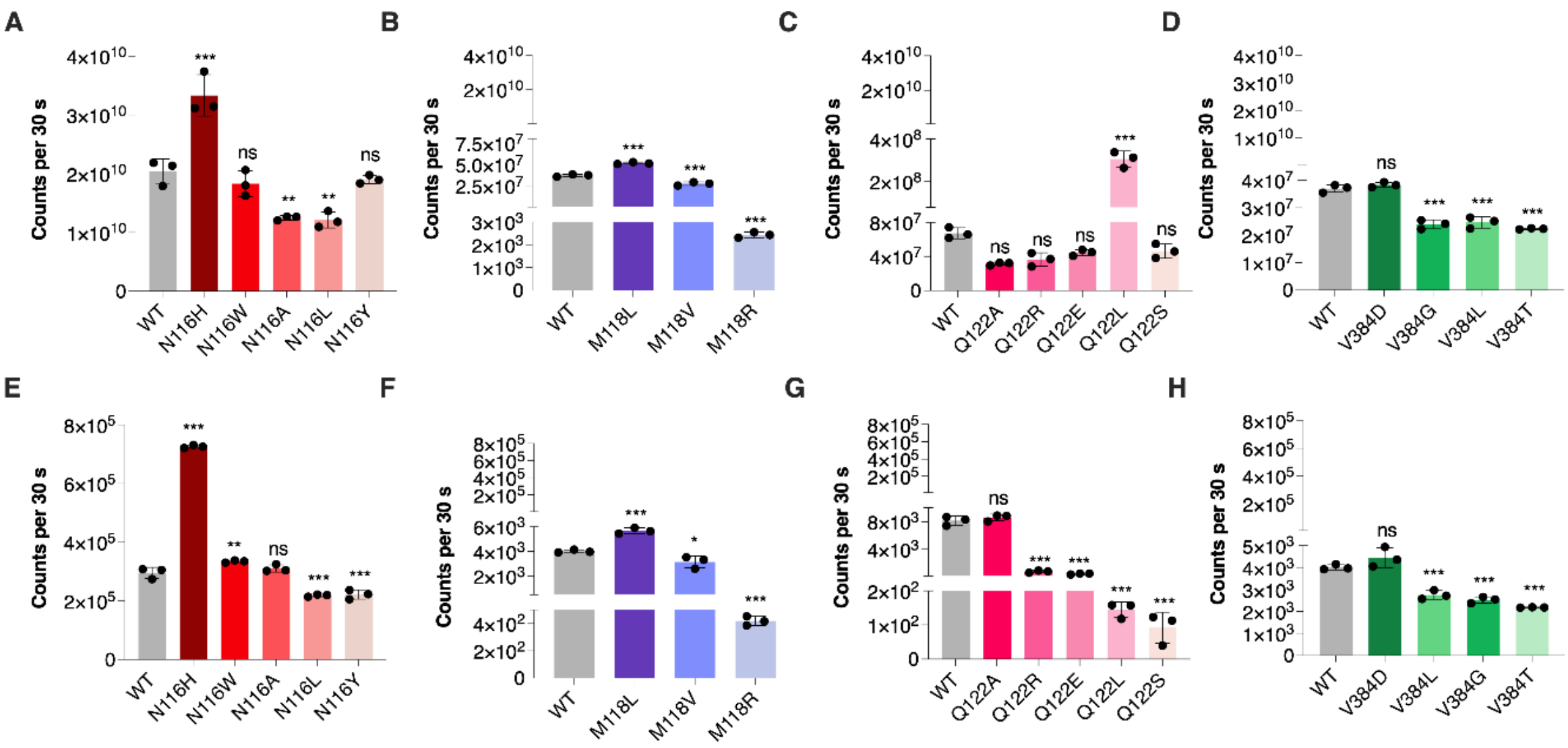

Fig. S4. Screening for thermostable Fluc variants using partially purified enzyme. **(A–D)** Activities of the partially purified (two-step precipitation by PEI and ammonium sulfate) Fluc variants from four libraries (**A**) N116X, (**B**) M118X, (**C**) Q122X, and (**D**) V384X. (**E–H**) Activities of the enzymes in **A–D** after heating at 45 °C for 20 min. Data are shown as mean ± S.D. (n=3). One-way ANOVA was used for statistical analysis; *$p < 0.01$, ** $p < 0.001$, *** $p < 0.0001$; ns = not significant.

A

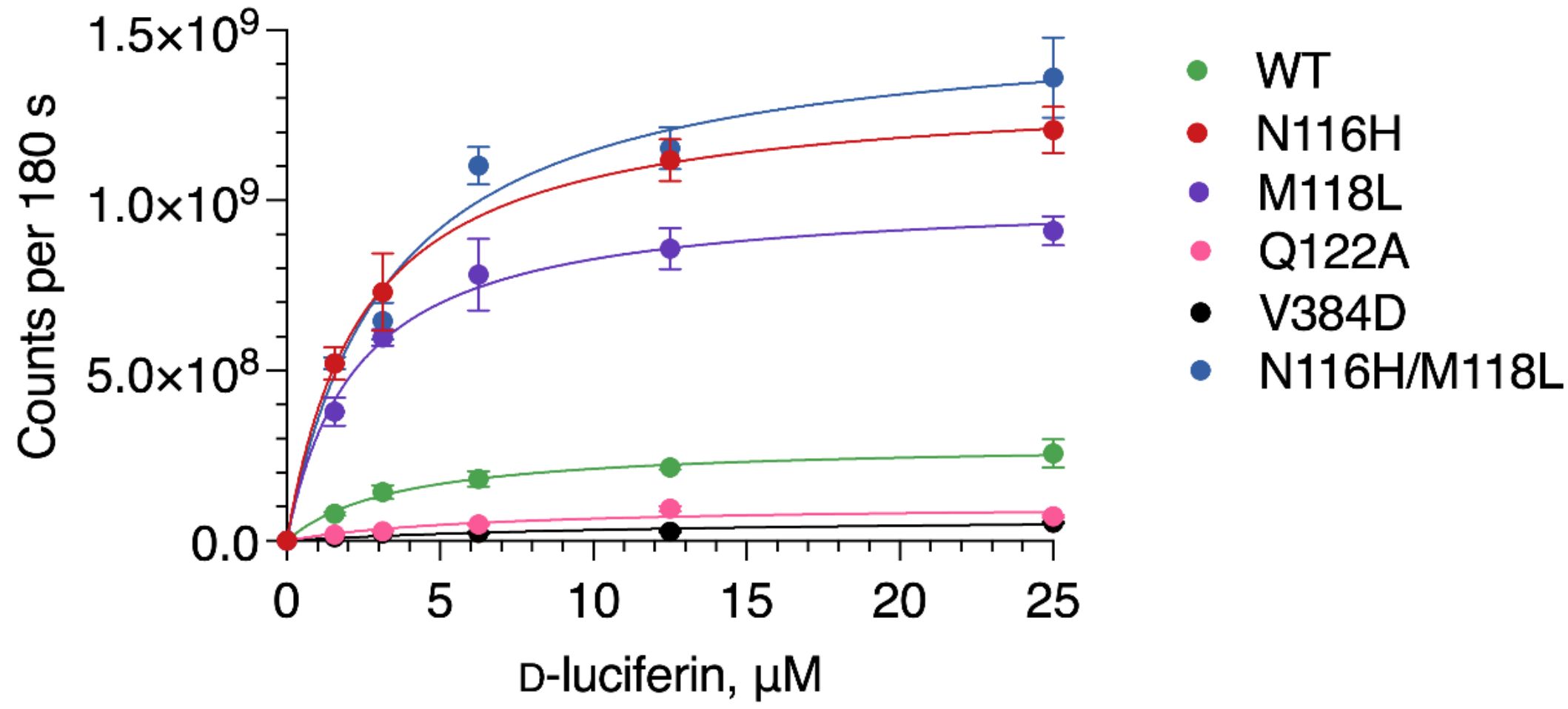

B

Enzyme	${}^{app}K_m$, µM	V_{max}, CPS	Relative k_{cat} (fold-change)
WT	3.64	1.60 x 10^6	1.0
N116H	2.49	7.40 x 10^6	4.6
M118L	2.28	5.65 x 10^6	3.5
Q122A	1.10	1.98 x 10^5	0.1
V384D	0.75	1.75 x 10^5	0.1
N116H/M118L	3.73	8.51 x 10^6	5.3

Fig. S5. Steady-state kinetics of Fluc variants. (**A**) plots of bioluminescence intensity for WT, N116H, M118L, Q122A, V384D, and N116H/M118L. (**B**) summary of kinetic parameters. Reactions (150 µL) were performed in 100 mM Tris-HCl buffer (pH 8.0) at 25 °C. Bioluminescence was initiated by adding 3 mM ATP and 6 mM $MgCl_2$ to 25 µL of purified luciferase preincubated with D-luciferin (0–50 µM). Signals were recorded for 180 s. Relative k_{cat} values were obtained by normalizing light output to enzyme concentration (15.625 nM), and kinetic parameters were determined by nonlinear regression using *GraphPad Prism* version 10.6.1. Data represent the mean ± S.D. (n=3).

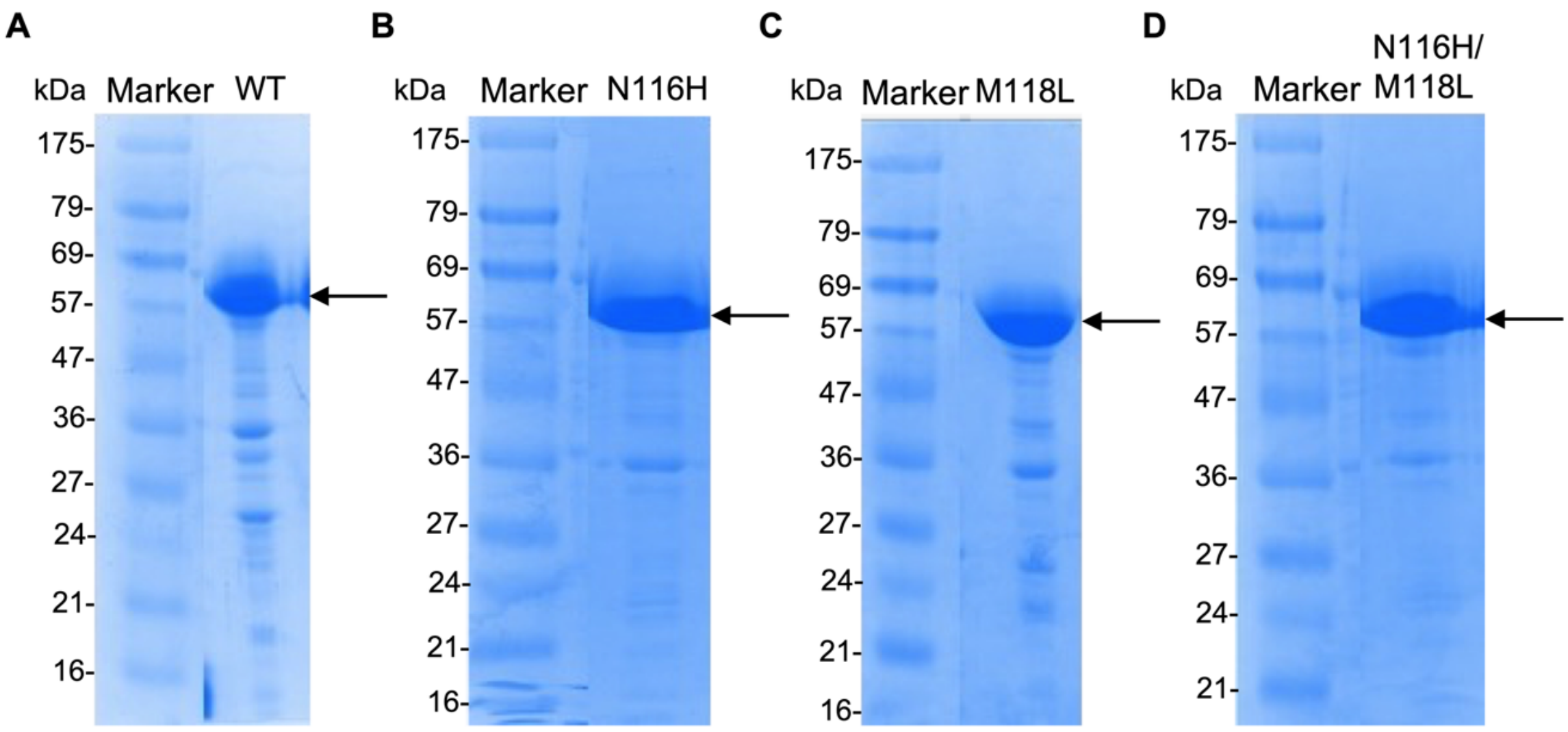

Fig. S6. 12% SDS-PAGE analysis of purified Fluc variants–(**A**) WT, (**B**) N116H, (**C**) M118L and (**D**) N116H/M118L. Twenty micrograms of proteins were loaded per lane. The arrows indicate the target protein at ~62 kDa.

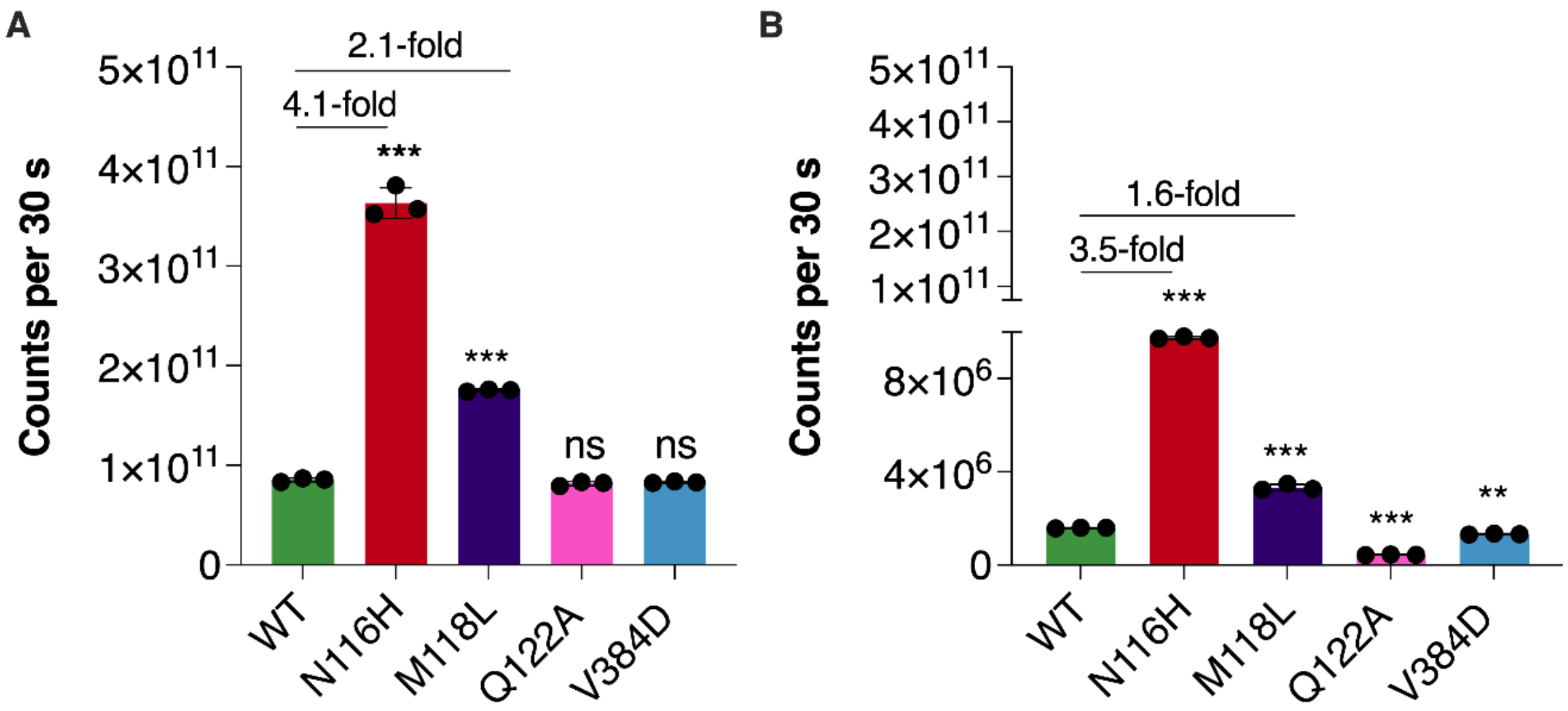

Fig. S7. Luciferase activities of purified Fluc variants (A) before and (B) after heat treatment at 45 °C for 20 min. Purified luciferase variants included WT, N116H, M118L, Q122A, and V384D were measured activities before and after heat treatment at 45 °C for 20 min. The concentration of non-preheated variants used for the assay was 4 nM. For the heat treatment, 10 µM enzyme was incubated at 45 °C for 20 min and centrifuged (2,700 x g for 10 min). The resulting 5 µL of the supernatant (equivalent to a final concentration of 4 nM) was used in a 125 µL assay reaction. Data shown are the mean ± S.D. (n=3). The data were analyzed using One-way ANOVA; ** $p < 0.001$, *** $p < 0.0001$; ns = not significant.

A

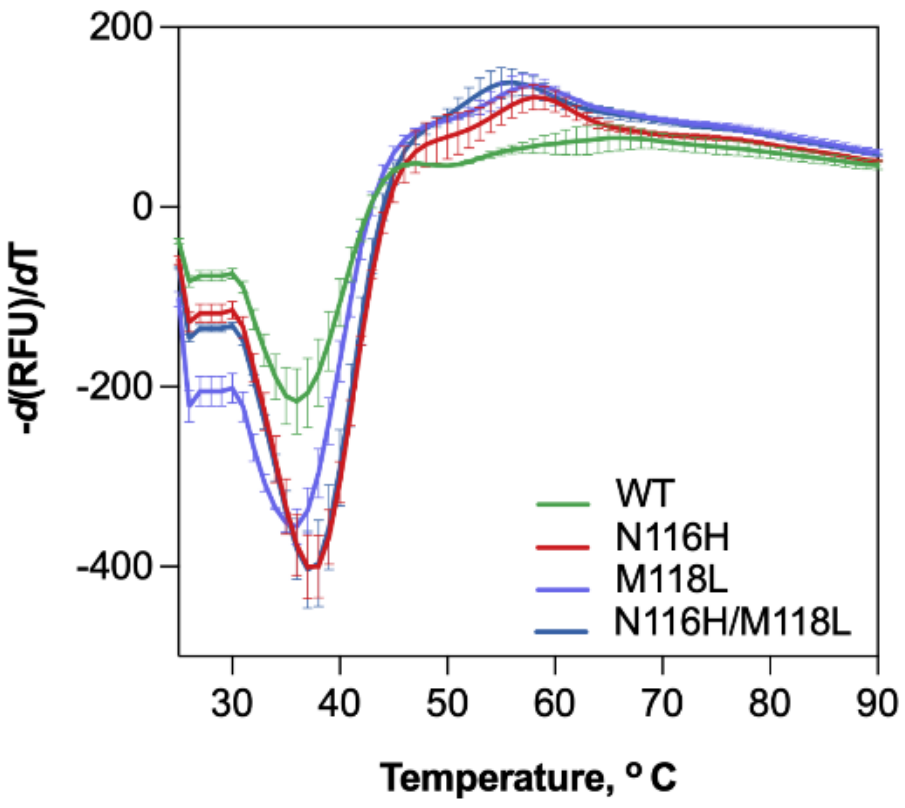

B

Enzymes	T_m, min	± S.D.
WT	36.0	0.0
N116H	38.0	0.0
M118L	35.7	0.6
N116H/M118L	37.0	0.0

C

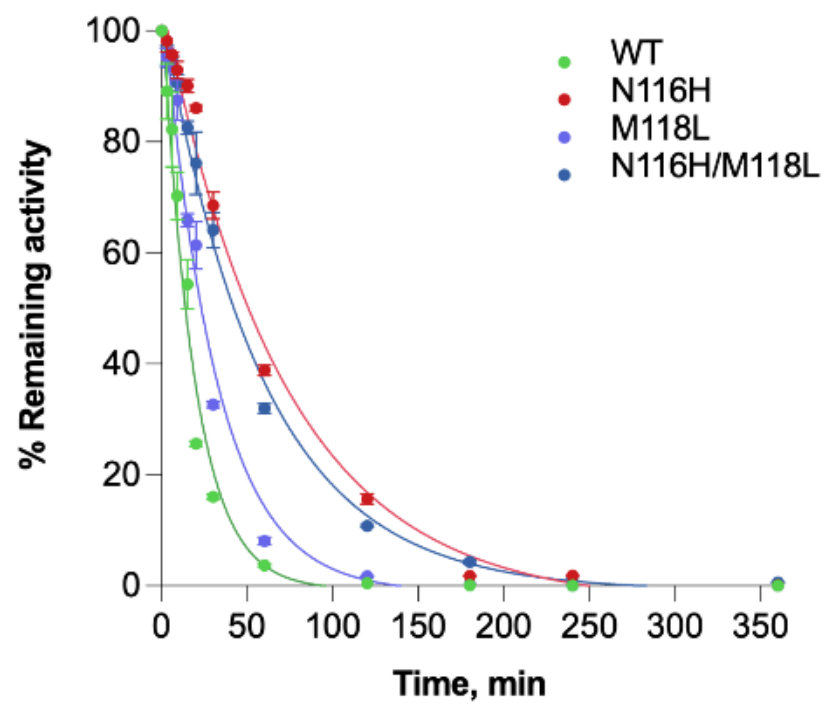

D

Enzymes	$^{37}t_{1/2}$, min	± S.D.	fold-change
WT	13	1.0	-
N116H	46	2.9	3.5
M118L	21	1.2	1.6
N116H/M118L	37	6.7	2.8

Fig. S8. Assessment of thermal stability of Fluc variants. (**A**) Derivative plots of fluorescence changes of the WT, N116H, M118L, and N116H/M118L variants upon temperature increase. (**B**) Summary of the T_m values of Fluc variants calculated from the inflection point of the curves in A. (**C**) Residual activities of Fluc variants (10 µM) during incubation at 37 °C for 6 h. The remaining activity (%) was calculated by normalizing the activity at each time point to the activity at time 0 (defined as 100%). The data were fitted to a one-phase exponential decay nonlinear regression model using *GraphPad Prism* 10.6.1 to estimate the $t_{1/2}$ of each variant. **(D)** Summary of the calculated $t_{1/2}$ values of Fluc variants. Data represent the mean ± S.D. (n=3).

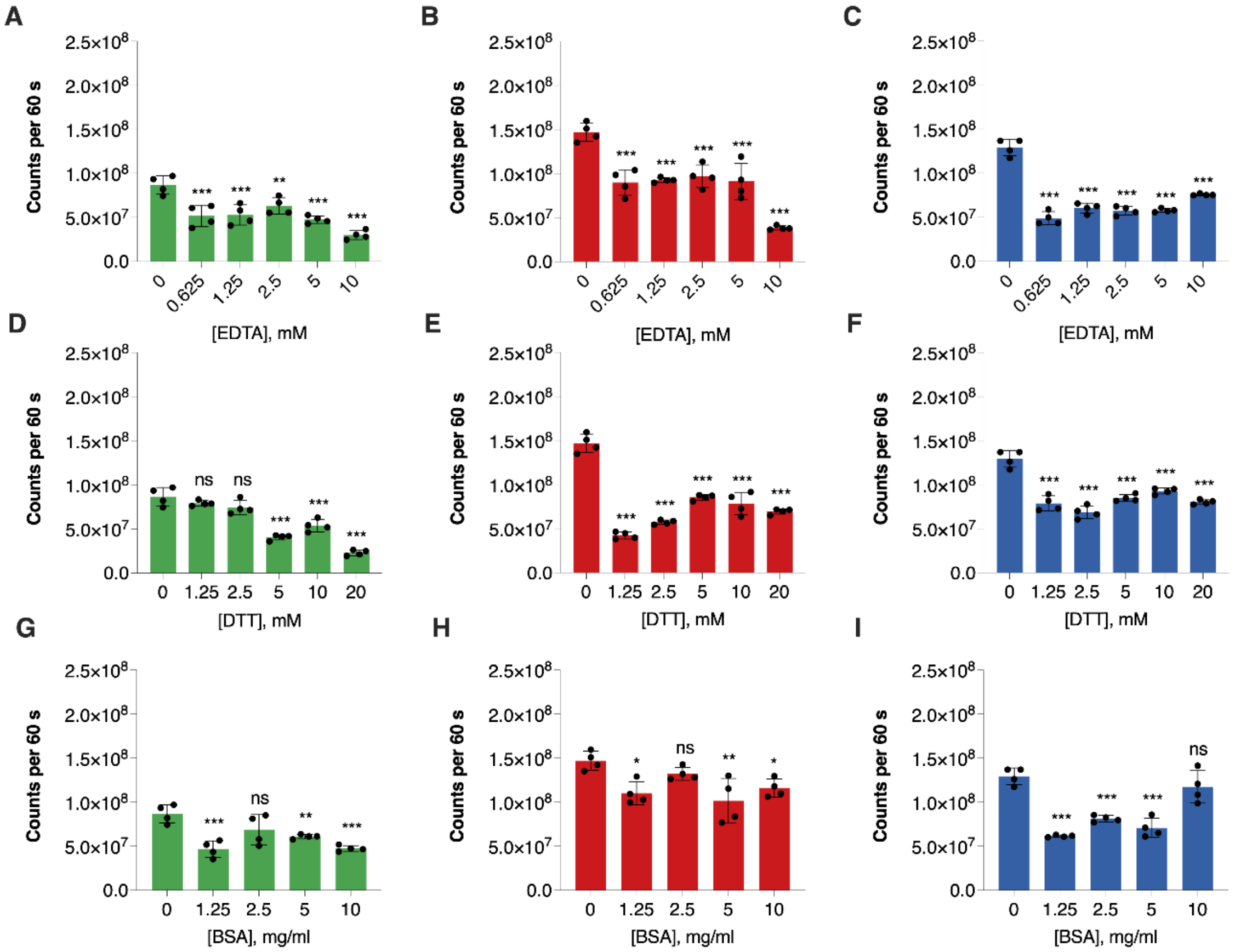

Fig. S9. Effects of additives–(A–C) EDTA, (D–F) DTT, and (G–I) BSA–on bioluminescence activities of WT (green), N116H (red), and N116H/M118L (blue) variants. Each purified variant (1 µM) was pre-mixed with individual additive at varying concentrations and incubated for 10 min before activity measurements. Data are presented as mean ± S.D. (n=4). One-way ANOVA was used for statistical analysis; *$p < 0.01$, ** $p < 0.001$, *** $p < 0.0001$; ns = not significant.

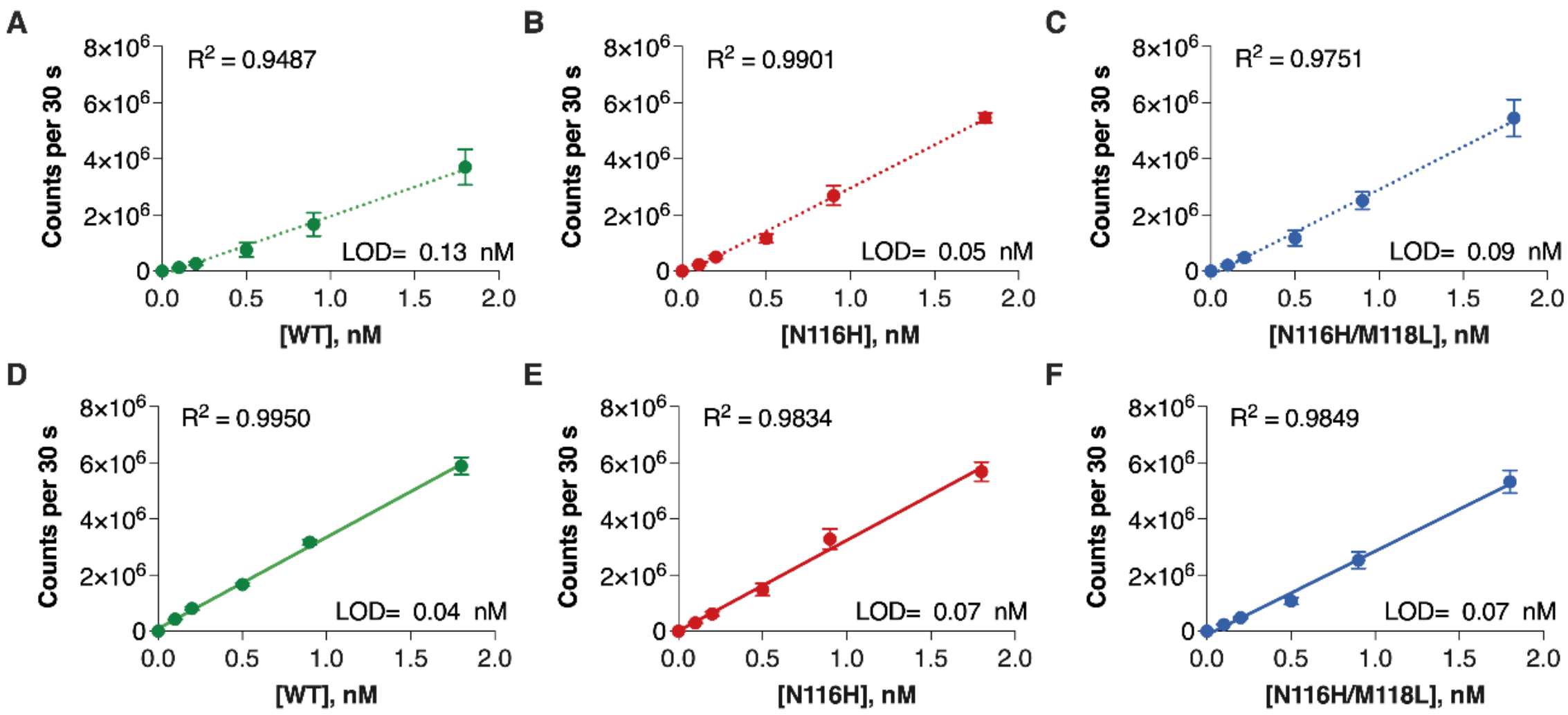

G

[CoA], mM	Fluc variant	Detection sensitivity (LOD)
	WT	0.13
0	N116H	0.05
	N116H/M118L	0.09
	WT	0.04
0.1	N116H	0.07
	N116H/M118L	0.07

Fig. S10. Correlation of bioluminescence signals with concentrations of Fluc variants. Bioluminescence assays were carried out in the absence (**A–C**) or presence of 0.1 mM CoA (**D–F**). (**G**) Summary of detection sensitivity of Fluc variants in the absence and presence of CoA. Data are shown as mean ± S.D. (n=3).

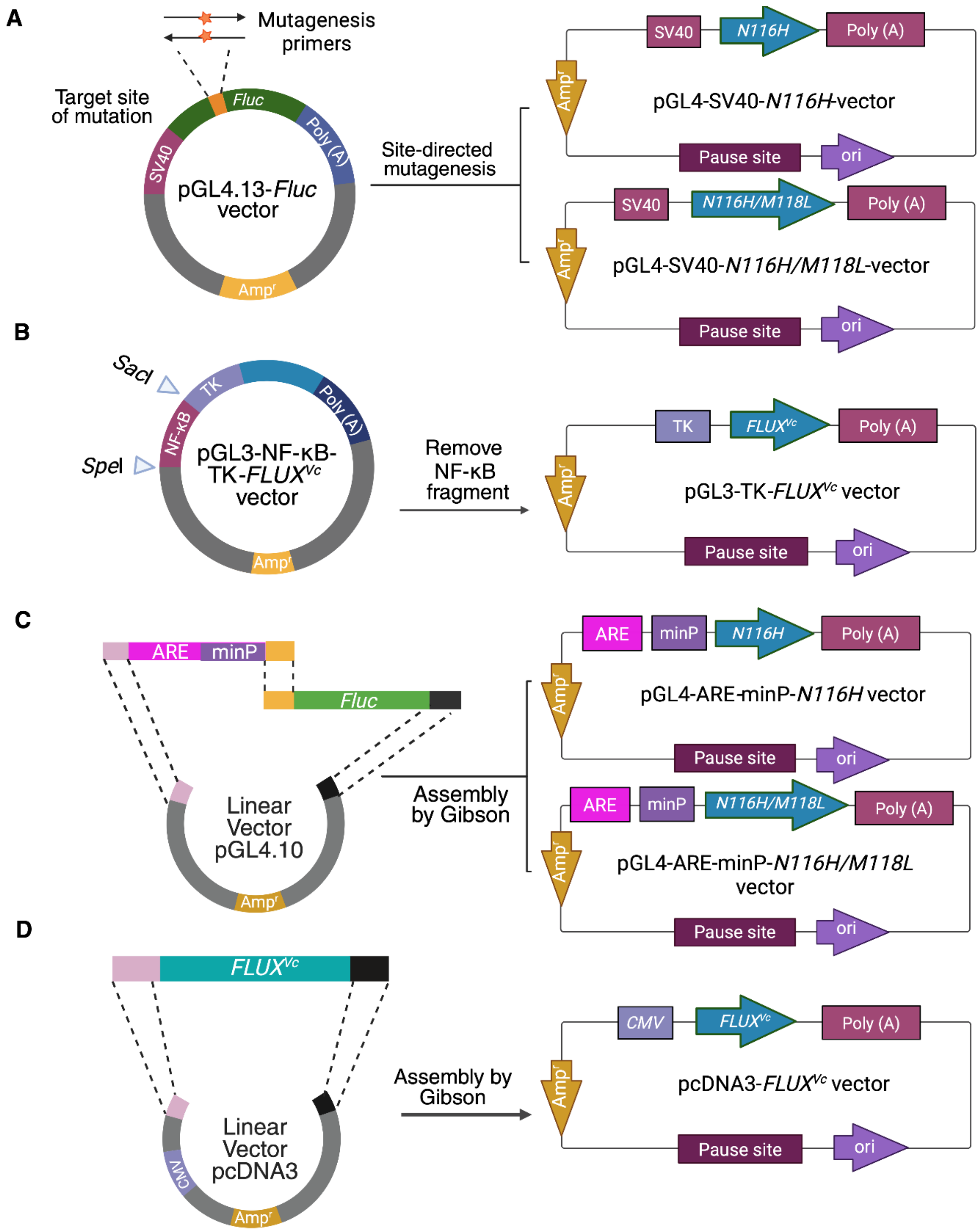

Fig. S11. Construction of mammalian cell expression plasmids used in this study. (**A**) pGL4-SV40-*Fluc* variants (N116H and N116H/M118L) were generated from pGL4 by site-directed mutagenesis using specific primers shown in **Table S2.** (**B**) pGL3-TK-*FLUX*^{Vc} (internal control vector) was constructed from pGL3-NF-κB-TK-*FLUX*^{Vc} (1)

by removing the NF-κB fragment *via* restriction digestion with *Sac*I and *Spe*I and followed by ligation using T4 DNA polymerase. (**C**) pcDNA3-*FLUX*^{Vc} (internal control vector) was generated by Gibson assembly of linearized pcDNA3 and PCR-amplified *FLUX*^{Vc} fragment. (**D**) pGL4-ARE-minP-*Fluc* variants were constructed by Gibson assembly of linearized pGL4.10, PCR-amplified ARE-minP, and *Fluc* variants (N116H and N116H/M118L).

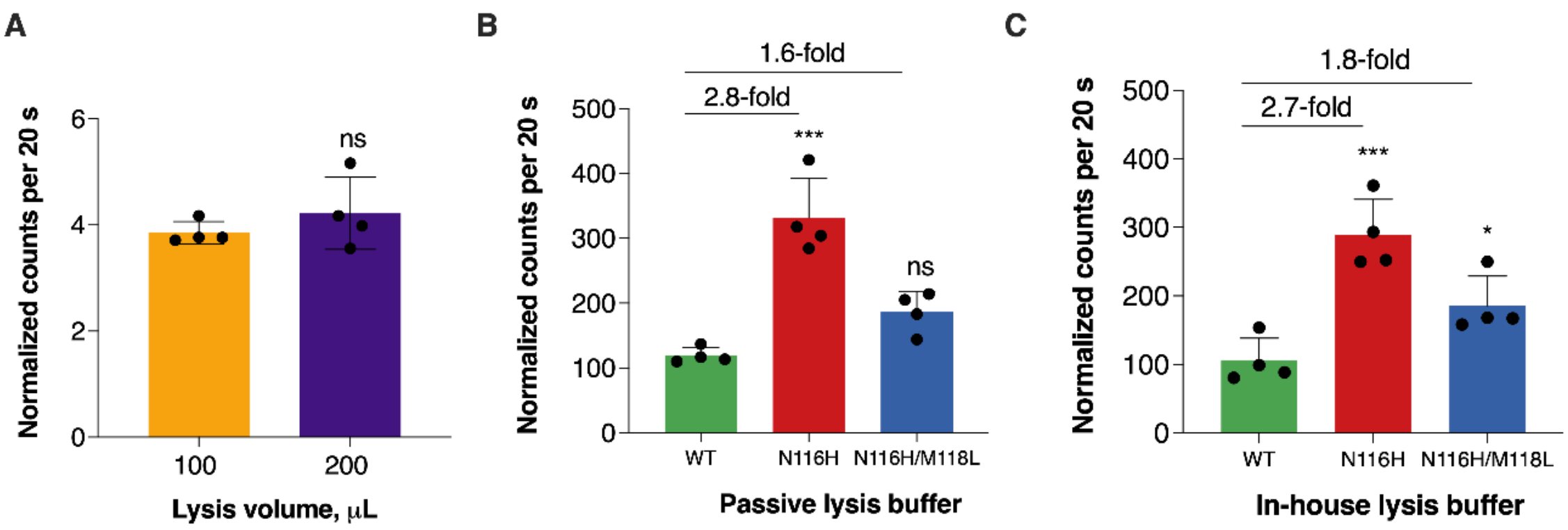

Fig. S12. Evaluation of in-house lysis reagents for use in assays of the combined Fluc/FLUX^{Vc} dual-reporter system. (**A**) Signal comparisons for the lysis of HEK293T cells co-transfected with pGL4-SV40-*Fluc* (WT) and pGL3-TK-*FLUX^{Vc}* using 100 and 200 μL in-house lysis buffer. Activities of Fluc and FLUX^{Vc} were measured and Fluc signals were normalized to FLUX^{Vc} activities. Data are shown as mean ± SD from (n=4). Statistical analysis was performed using Student's t-test. (**B–C**) Signal comparisons for the lysis of HEK293T cells co-transfected with pGL4-SV40-*Fluc* (WT), N116H, or N116H/M118L variants along with pGL3-TK-*FLUX^{Vc}* using (**B**) Passive Lysis Buffer (Promega) or (**C**) in-house lysis buffer. Data are shown as mean ± SD (n=4). One-way ANOVA was used for statistical analysis; *$p < 0.01$, *** $p < 0.0001$; ns = not significant.

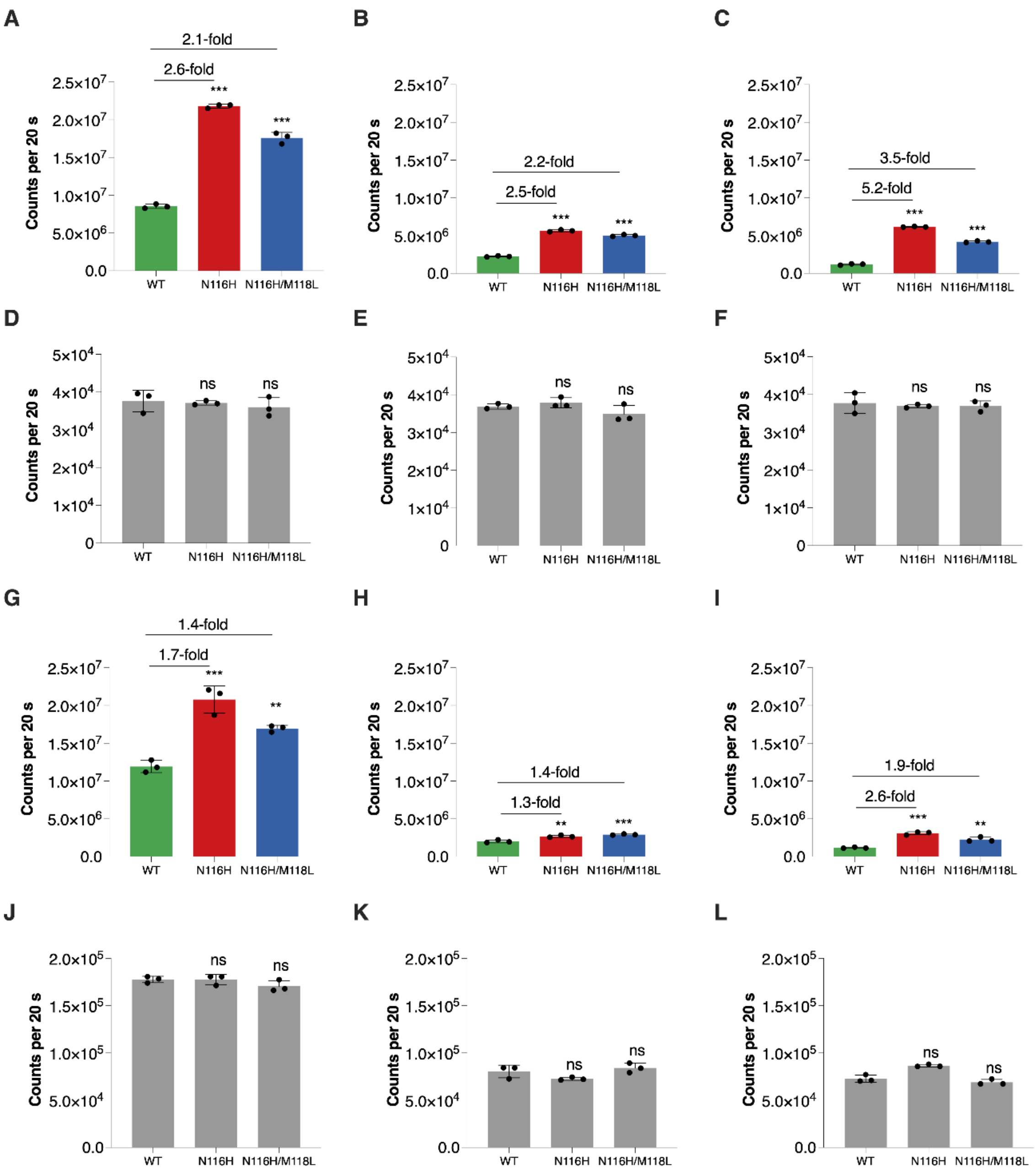

Fig. S13. Comparison of bioluminescence signals from WT and Fluc variants expressed in different cell lines using FLUX^{Vc} and Rluc as internal controls. Raw luciferase activities in lysates of HEK293T (**A, D, G, J**), HepG2 (**B, E, H, K**), and HeLa (**C, F, I, L**) cells were measured. (**A–C**) Raw Fluc activities when co-transfected with FLUX^{Vc}. (**D–F**) Raw FLUX^{Vc} internal control activity. (**G–I**) Raw Fluc activities when co-transfected with Rluc. (**J–L**) Raw Rluc internal control activities. Data represent mean

± SD (n = 3). Statistical significance was determined by one-way ANOVA: ** $p < 0.001$, *** $p < 0.0001$; ns = not significant.

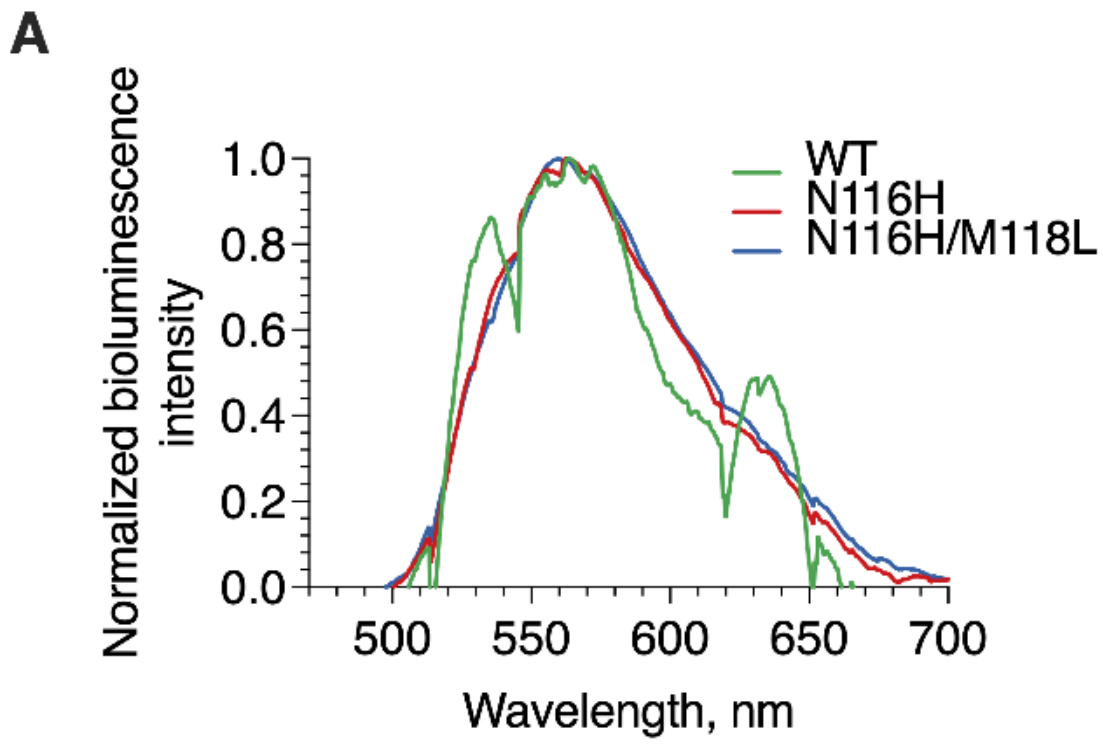

B

Fluc variant	$^{BL}\lambda_{max}$, nm
WT	565
N116H	565
N116H/M118L	565

Fig. S14. Bioluminescence emission spectra from HepG2 expressing various Fluc variants. (A) Emission spectra from HepG2 cells expressing WT (green), N116H (red), and N116H/M118L (blue). (**B**) Maximum emission wavelengths of Fluc variants. Bioluminescence was recorded using a highly sensitive CCD spectrophotometer (ATTO AB-1850s, Lumi Fluoro Capture) with 5 s shutter time and 1 mm slit width. Data were smoothed using *GraphPad Prism* 10.6.1.

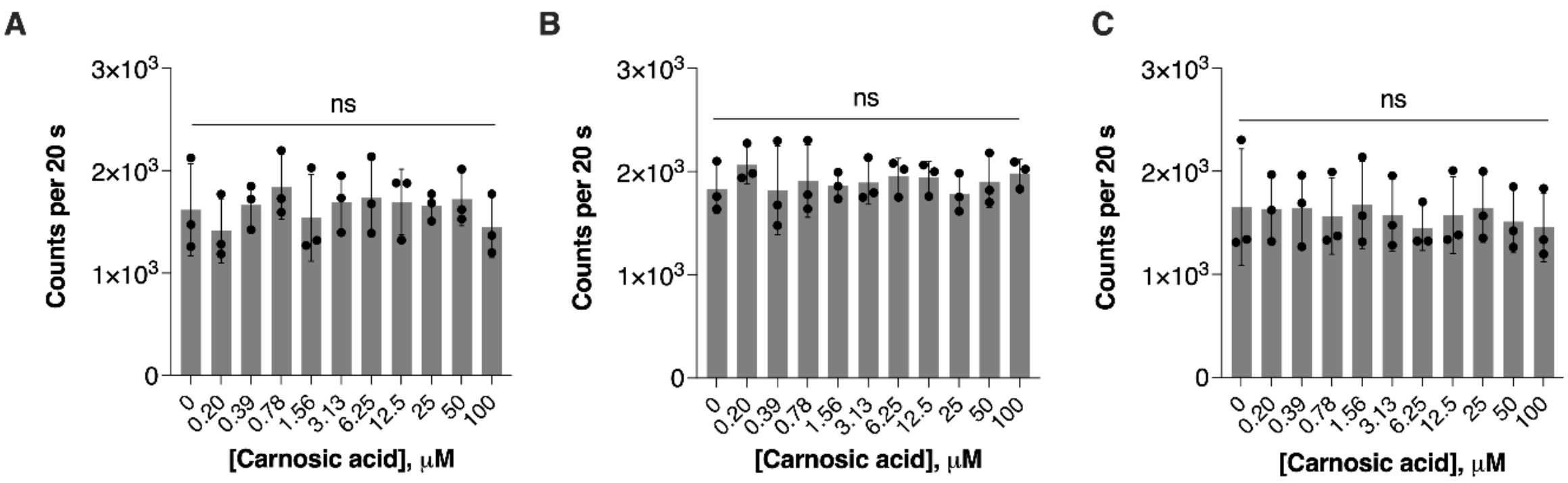

Fig. S15. Bioluminescence activities of HepG2 cells co-transfected with bacterial luciferase (FLUX^{Vc}) and ARE-*Fluc* constructs in the presence of varying carnosic acid concentrations. Fluc variants are (**A**) WT, (**B**) N116H, and (**C**) N116H/M118L. Data shown are mean ± SD (n= 3). Significance determined by one-way ANOVA using *GraphPad Prism* 10.6.1: ns = not significant.

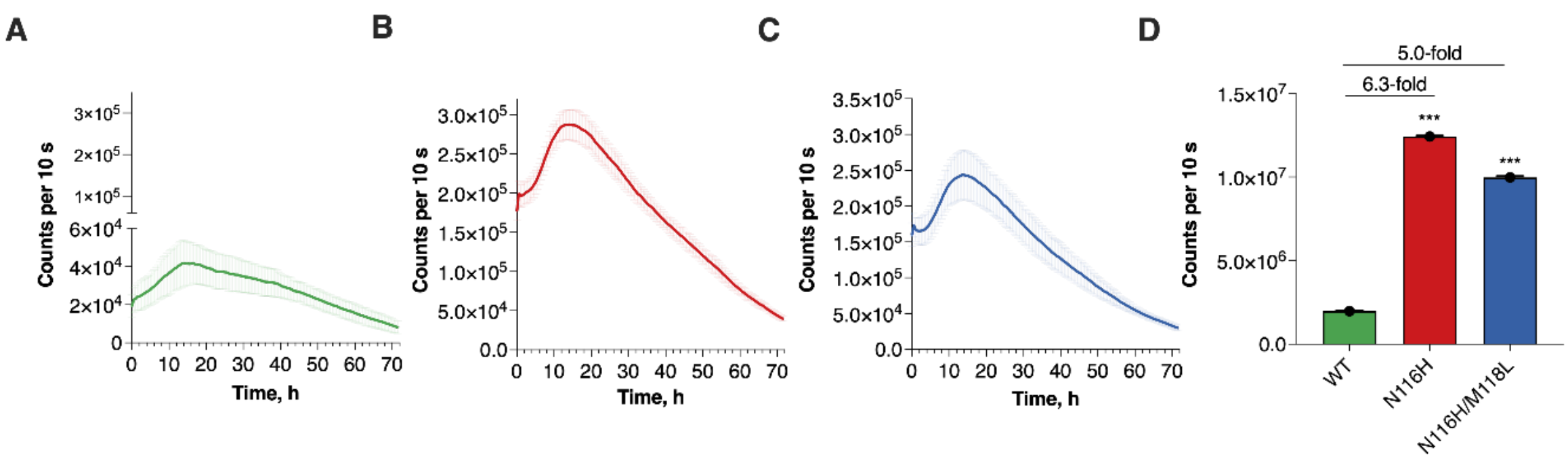

Fig. S16. Real-time bioluminescence kinetics of HepG2 cells expressing WT and Fluc variants–(**A**) WT, (**B**) N116H, (**C**) N116H/M118L. Bioluminescence (10 s for each collection) was recorded every 30 min intervals over 72 h using a WSL-1565 Kronos-HT (ATTO). Data are shown as mean counts ± SD (per 10 s; n=4). (**D**) Comparisons of bioluminescence intensity calculated from the area under the curve (AUC) from the

bioluminescence kinetics. Statistical significance was determined by one-way ANOVA: ***$p < 0.001$.

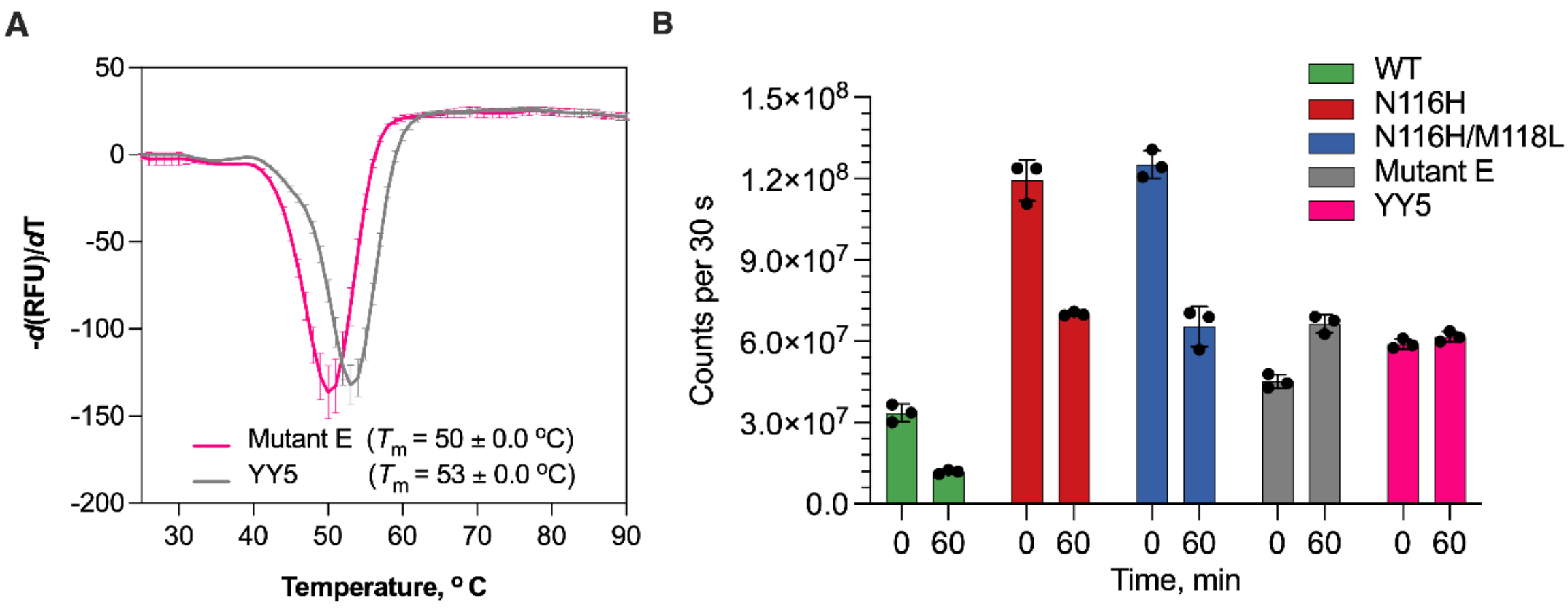

Fig. S17. Thermal stability of Fluc variants. (**A**) Derivative plots of temperature and fluorescence of the Mutant E and YY5 variants. (**B**) Purified luciferase variants included WT, N116H, N116H/M118L, Mutant E, and YY5 were measured activities before and after heat treatment at 37 °C for 60 min. A concentration of non-preheated variants used for the assay was 4 nM. For the heat treatment, 0.5 µM enzyme were incubated at 37 °C for 60 min and centrifuged (2,700 x g for 10 min). The resulting 5 µL of the treated enzyme solution (equivalent to a final concentration of 4 nM) was used in a 125 µL assay reaction. Data shown are the mean ± S.D. (n=3).

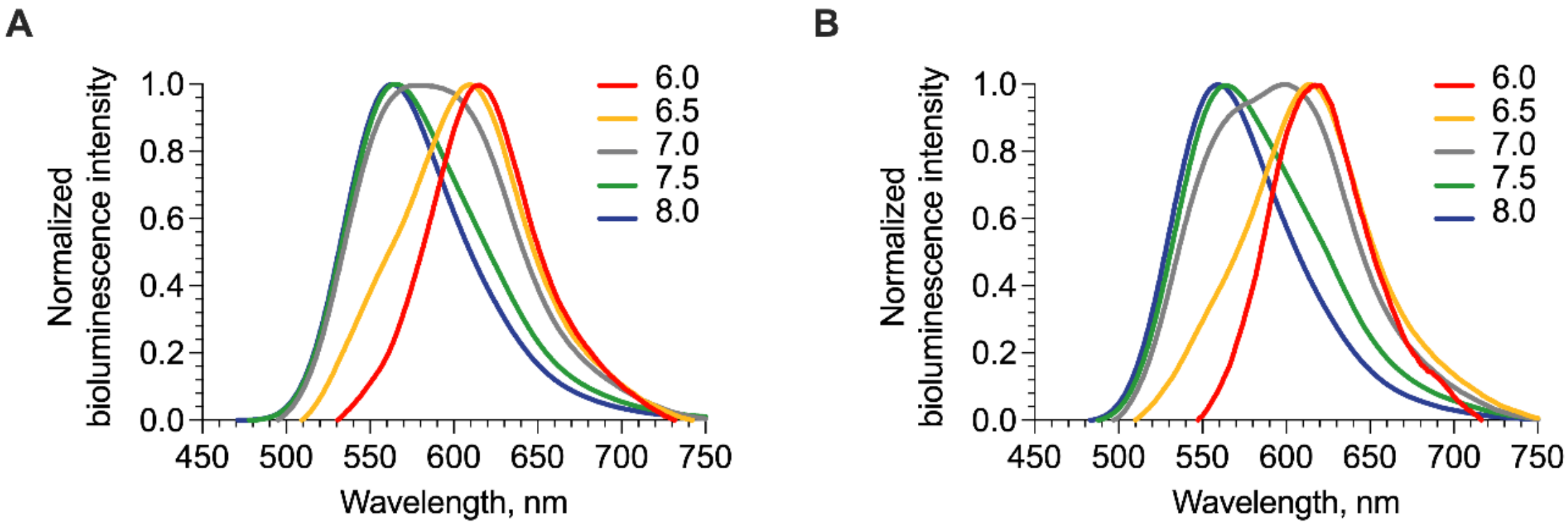

C

pH	WT $^{BL}\lambda_{max}$, nm	N116H $^{BL}\lambda_{max}$, nm
6.0	614.7 ±1.6	617.6 ± 2.1
6.5	609.5 ±1.4	613.8 ±1.2
7.0	587.6 ±6.8	600.0 ±0.5
7.5	565.1 ±0.5	564.6 ±3.7
8.0	563.0 ±0.5	559.6 ±1.7

Fig. S18. pH-dependent bioluminescence emission spectra of WT and Fluc-N116H variants. Bioluminescence emission spectra of purified WT (**A**) and N116H (**B**) Fluc were measured across pH 6.0–8.0 using a tri-buffer system (40 mM MES, 40 mM Tris, and 40 mM sodium phosphate) with the indicated pH. Reactions contained 3 mM ATP, 6 mM $MgCl_2$, 20 µM D-LH_2, and 60 nM purified enzyme in a final volume of 150 µL. Spectra were recorded immediately after enzyme addition with a shutter time of 10 s and a slit width of 1 mm. The data were normalized to their respective maximum emission intensities. (**C**) Summary of bioluminescence emission maxima ($^{BL}\lambda_{max}$) for WT and N116H Fluc at each pH tested. Data represent mean ± SD. (n=3).

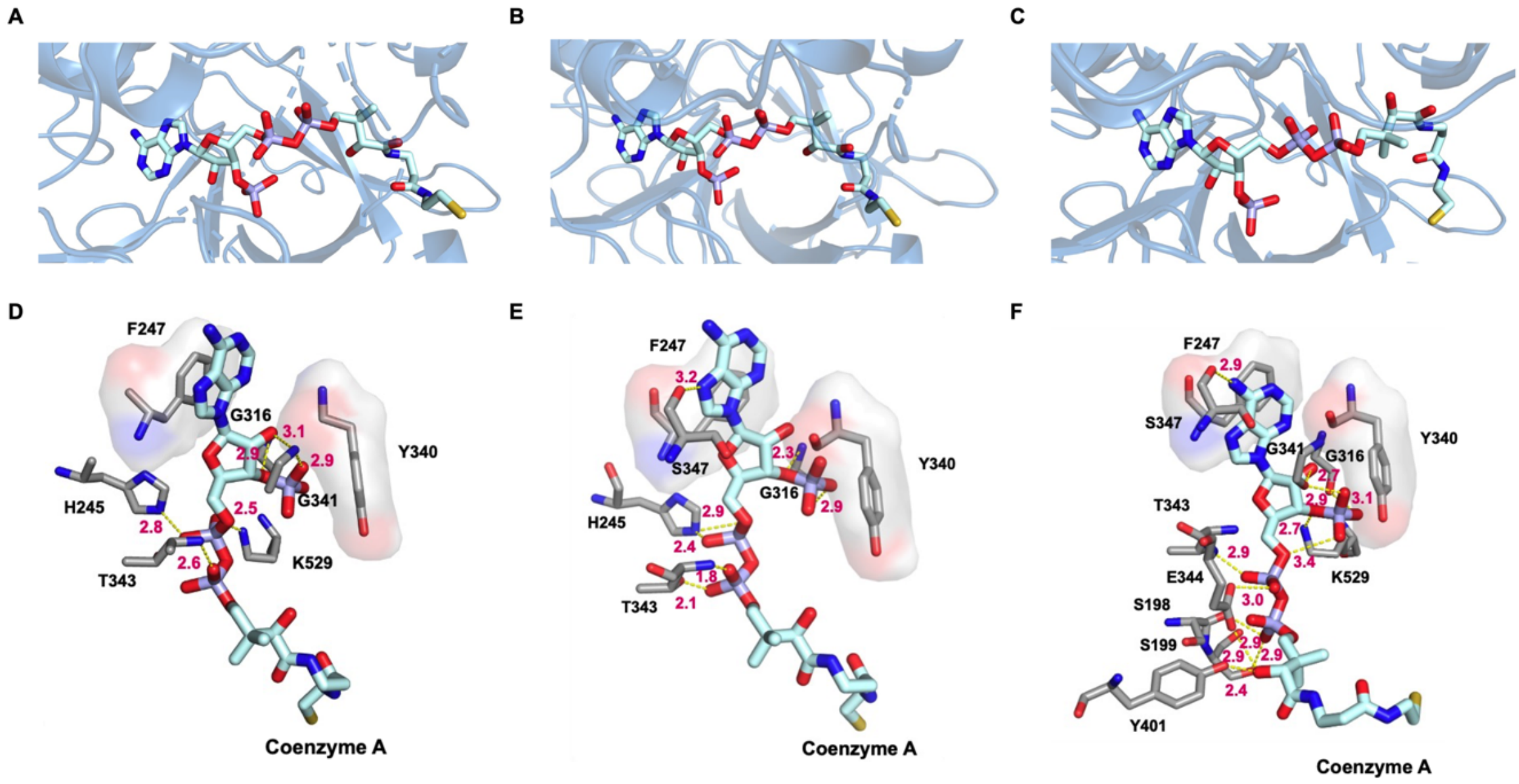

Fig. S19. Predicted CoA-binding mode in WT and Fluc variants. Predicted CoA-binding modes in WT and Fluc variants were investigated by molecular docking**.** (**A–C**) Predicted CoA-binding sites for WT, N116H, and N116H/M118, respectively. The adenylate-forming conformation was generated using the WT Fluc structure in complex with 5′-O-[N-(dehydroluciferyl)-sulfamoyl] adenosine (DLSA) (PDB ID: 4G36) (2) as the starting model. *In silico* mutations were performed using CHARMM-GUI (3) prior to docking. Docking was performed with GOLD (4), and the top-ranked configuration was selected based on the highest Piecewise Linear Potential (PLP) fitness score. (**D–F**) Enlarged views of the predicted CoA-binding sites for WT, N116H, and N116H/M118L, respectively, show a similar CoA-binding orientation across all three enzymes. Images were prepared using PyMOL 2.5 (Schrödinger) (5). Atom colors: oxygen, red; nitrogen, dark blue; sulfur, yellow; phosphorus, purple; protein carbon, gray; CoA carbon, cyan.

Table S1. List of 36 Fluc variants predicted for thermostability improvement by FireProt (6, 7). Surface residues were selected for site-directed mutagenesis. (√) surface residues, (-) non-surface residues. The M30L and Y266P variants were excluded because their PCR products could not be successfully constructed at the beginning of the study.

No.	Mutation	FoldX (kcal/mol)	Surface residue	No.	Mutation	FoldX (kcal/mol)	Surface residue
1	M30L	0.16	√	19	S239I	-4.11	-
2	E48G	-0.76	√	20	H244F	-2.07	-
3	M67L	-0.49	√	21	G246A	-1.40	-
4	N73K	0.33	√	22	M265L	0.41	√
5	V80I	-0.49	-	23	Y266P	-0.24	√
6	C81F	-1.24	-	24	S284Q	-1.12	-
7	Q87W	-2.41	√	25	S293V	0.37	√
8	D107P	-3.93	-	26	A313L	-2.71	-
9	N116H	-0.12	√	27	S314C	0.04	-
10	S117A	-0.45	√	28	G316A	-1.60	-
11	M118L	0.39	√	29	G324A	-1.46	√
12	Q122K	-1.82	√	30	A348F	-1.71	-
13	T124W	-1.17	√	31	T352M	-1.09	-
14	Q134P	-1.53	√	32	A361S	-0.70	√
15	M152L	0.38	√	33	V384P	-0.77	√
16	N197C	0.04	-	34	S399K	-0.42	√
17	N229W	-1.17	-	35	S420T	-1.80	√
18	Q230P	-1.13	√	36	W426Y	-0.76	√

Table S2. List of Primers used for construction of site-saturation variants libraries and mammalian expression vectors. The substituted codons are marked in bold.

**Primer**	**Nucleotide sequence (5'-3')** [a]
N116X_F	GTGAACTTCTT**NNK**AGTATGGGTATTTCCCAACCGACCGT
N116X_R	ATACCCATACT**MNN**AAGAAGTTCACGCTCATTATATATGT
M118X_F	TTCTTAATAGT**NNK**GGTATTTCCCAACCGACCGTGGTTTT
M118X_R	TGGGAAATACC**MNN**ACTATTAAGAAGTTCACGCTCATTAT
Q122X_F	TGGGTATTTCC**NNK**CCGACCGTGGTTTTTGTTTCGAAAAA
Q122X_R	ACCACGGTCGG**MNN**GGAAATACCCATACTATTAAGAAGTT
V384X_F	AGACCTTGGGT**NNK**AACCAACGGGGGGAATTGTGTGTACG
V384X_R	CCCCGTTGGTT**MNN**ACCCAAGGTCTTACCCGTATCTAAAT
N116H-*Fluc*_F	AGCTGCTG**CAT**AGCATGGGCATCAGC
N116H-*Fluc*_R	GCCCATGCT**ATG**CAGCAGCTCGCGCTC
N116H/M118L-*Flu*c_F	AGCTGCTG**CAT**AGC**CTT**GGCATCAGCCAGCCC
N116H/M118L-*Flu*c_R	TGATGCC**AAG**GCT**ATG**CAGCAGCTCGCGCTCG
pcDNA3-MSC_F	TGCTGGAATTCTGCAGATATCCA
pcDNA3-MSC_R	AGTGGATCCGAGCTCGGTAC
FLUX^{Vc}-pcDNA3_F	GAGCTCGGATCCACTGCCACCATGGGCATGAAG
FLUX^{Vc}-pcDNA3_R	TGCAGAATTCCAGCATCATGAGTGGTATTTCACGATATTT

X indicates randomly site-saturation mutagenesis.

[a] N is A, T, C, or G; K is G or T; M is A or C.

Table S3. Preparation and cost estimation for in-house luciferase assay reagents.

Buffer type	Component	Final concentration	Amount per 1 L	Vendor	Estimated price per 1L (USD)	Total cost (USD)
**Lysis buffer** (200 µL / assay)	CHAPS		10.0 g	Sigma	90.00	95.0
	Glycerol	10% (v/v)	100 mL	Standard lab stock	5.00	
	EDTA	1 mM	~0.37 g	Standard lab stock	< 0.01	
**Firefly assay** (100 µL / assay)	Tris-HCl	100 mM	~12.1 g (Tris base)	Standard lab stock	1.00	50.02
	ATP (Disodium)	3 mM	1.65 g	Thermo Fisher	41.25	
	$MgCl_2$	6 mM	1.22 g	Standard lab stock	1.00	
	D-LH_2	25 µM	8.0 mg	Promega	6.77	
**Bacterial assay** (100 µL / assay)	Na_2HPO_4/NaH_2PO_4	50 mM	~10.6 g (mixed)	Standard lab stock	0.50	187.04
	FMN	5 µM	2.39 mg	Sigma-Aldrich	0.27	
	NADH	100 µM	70.9 mg	Sigma-Aldrich	41.12	
	Decanal	10 µM	1.56 mg (~1.9 µL)	Sigma-Aldrich	< 0.01	
	HPA	100 µM	15.2 mg	Sigma-Aldrich	0.14	
	C1 reductase	0.5 U/mL	500 Units	Biosynthai	145.00	

Table S4. Cost comparison per assay (in-house formulations vs. commercial kit)

Assay component	**In-House Formulation (USD)**	**Promega commercial equivalent (USD)***
Cell lysis (200 µL)	0.019	0.290 *(Passive Lysis Buffer)*
Target reporter: Fluc assay (100 µL)	0.005	0.900–2.660 *(Luciferase Assay System)*
Control reporter: Flux/Rluc (100 µL)	0.019 *(Bacterial luciferase assay system)*	0.910–3.500 *(Renilla luciferase assay system)*
**Total cost per co-transfected well**	0.043	2.100–6.450

*Commercial equivalent costs are estimated ranges based on standard vendor list prices for standalone kits (Cat. #E1941, #E1500/#E1501, and #E2810/#E2820), depending on 100-assay *versus* 1,000-assay bulk purchasing tiers.

Table S5. Functional analysis of Fluc variants compared to WT in HepG2 cells.

Variant	Bioluminescence (BL) emission kinetics		Single cell analysis		
	AUC	AUC (fold-change)	Detected cell no.	BL /pixel	BL/pixel (fold-change)
**WT**	0.2×10^7	1.0	17	273.2	1.0
**N116H**	1.2×10^7	6.3	77	385.9	1.4
**N116H /M118L**	1.0×10^7	5.0	83	356.9	1.3

References

1. Phonbuppha, J., Tinikul, R., Ohmiya, Y., andChaiyen, P. (2023) High sensitivity and low-cost flavin luciferase (FLUXVc)-based reporter gene for mammalian cell expression Journal of Biological Chemistry **299**,
2. Sundlov, J. A., Fontaine, D. M., Southworth, T. L., Branchini, B. R., andGulick, A. M. (2012) Crystal structure of firefly luciferase in a second catalytic conformation supports a domain alternation mechanism Biochemistry **51**, 6493-6495 10.1021/bi300934s
3. Jo, S., Kim, T., Iyer, V. G., andIm, W. (2008) CHARMM-GUI: a web-based graphical user interface for CHARMM J Comput Chem **29**, 1859-1865 10.1002/jcc.20945
4. Verdonk, M. L., Cole, J. C., Hartshorn, M. J., Murray, C. W., andTaylor, R. D. (2003) Improved protein-ligand docking using GOLD Proteins **52**, 609-623 10.1002/prot.10465
5. Schrödinger, L. (2021) The PyMOL Molecular Graphics System **2.5**, https://pymol.org
6. Bednar, D., Beerens, K., Sebestova, E., Bendl, J., Khare, S., Chaloupkova, R. *et al.* (2015) FireProt: Energy- and Evolution-Based Computational Design of Thermostable Multiple-Point Mutants PLoS Comput Biol **11**, e1004556 10.1371/journal.pcbi.1004556
7. Musil, M., Stourac, J., Bendl, J., Brezovsky, J., Prokop, Z., Zendulka, J. *et al.* (2017) FireProt: web server for automated design of thermostable proteins Nucleic Acids Res **45**, W393-w399 10.1093/nar/gkx285
